# Structural and mechanistic basis for translation inhibition by macrolide and ketolide antibiotics

**DOI:** 10.1038/s41467-021-24674-9

**Published:** 2021-07-22

**Authors:** Bertrand Beckert, Elodie C. Leroy, Shanmugapriya Sothiselvam, Lars V. Bock, Maxim S. Svetlov, Michael Graf, Stefan Arenz, Maha Abdelshahid, Britta Seip, Helmut Grubmüller, Alexander S. Mankin, C. Axel Innis, Nora Vázquez-Laslop, Daniel N. Wilson

**Affiliations:** 1 Institute for Biochemistry and Molecular Biology, University of Hamburg, Hamburg, Germany; 2 Univ. Bordeaux, Centre National de la Recherche Scientifique, Institut National de la Santé et de la Recherche Médicale, ARNA, UMR 5320, U1212, Institut Européen de Chimie et Biologie, Pessac, France; 3 Center for Biomolecular Sciences, University of Illinois at Chicago, Chicago, IL USA; 4 Theoretical and Computational Biophysics Department, Max Planck Institute for Biophysical Chemistry, Göttingen, Germany

**Keywords:** Antibiotics, Ribosome, Cryoelectron microscopy

## Abstract

Macrolides and ketolides comprise a family of clinically important antibiotics that inhibit protein synthesis by binding within the exit tunnel of the bacterial ribosome. While these antibiotics are known to interrupt translation at specific sequence motifs, with ketolides predominantly stalling at Arg/Lys-X-Arg/Lys motifs and macrolides displaying a broader specificity, a structural basis for their context-specific action has been lacking. Here, we present structures of ribosomes arrested during the synthesis of an Arg-Leu-Arg sequence by the macrolide erythromycin (ERY) and the ketolide telithromycin (TEL). Together with deep mutagenesis and molecular dynamics simulations, the structures reveal how ERY and TEL interplay with the Arg-Leu-Arg motif to induce translational arrest and illuminate the basis for the less stringent sequence-specific action of ERY over TEL. Because programmed stalling at the Arg/Lys-X-Arg/Lys motifs is used to activate expression of antibiotic resistance genes, our study also provides important insights for future development of improved macrolide antibiotics.

## Abstract (web summary)

Macrolides and ketolides antibiotics selectively interfere with the translation of a specific subset of proteins. Here the authors show how the macrolide erythromycin and the ketolide telithromycin interplay with the nascent polypeptide chain to arrest translation.

## Introduction

**Fig. 1:**
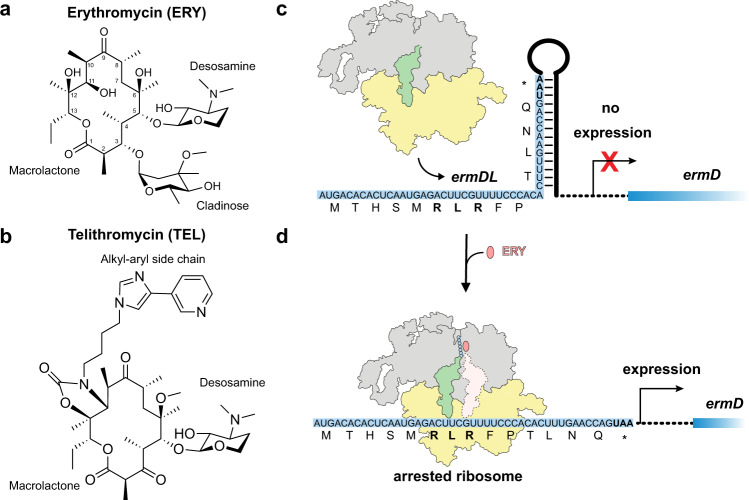
Macrolide-dependent regulation of *ermD* expression. **a**, **b** Chemical structures of **a** erythromycin (ERY) and **b** telithromycin (TEL). **c**, **d** Schematic of the *ermDL*-mediated regulation of the expression of *ermD* in the **c** absence and **d** presence of macrolide antibiotic.

The ribosome and protein synthesis represent one of the major targets in the bacterial cell for clinically-relevant antibiotics^1,2^. One important family of ribosome-targeting antibiotics are the macrolides, which display broad-spectrum activity against many Gram-positive bacteria, and have been in clinical usage since the discovery of the founding member erythromycin (ERY) in the 1950s^3,4^. ERY contains a 14-membered macrolactone ring and is decorated with cladinose and desosamine sugars at the C3 and C5 positions, respectively Fig. 1a). The emergence of antibiotic resistance to ERY and other macrolide antibiotics prompted the development of semi-synthetic derivatives, including the third generation ketolides, such as telithromycin (TEL)^3,4^. Like ERY, TEL contains a 14-membered macrolactone ring and the C5-desosamine, but lacks the C3-cladinose, which is replaced with a keto group (hence the name ketolide) (Fig. 1b). Additionally, TEL contains an extended alkyl-aryl side chain (Fig. 1c) linked via a carbamate to the C11 and C12 of the macrolactone, which is important for its bactericidal activity^5,6^. Structures of macrolides and ketolides in complex with the ribosome revealed that these compounds bind in a similar fashion within the nascent polypeptide exit tunnel (NPET)^7–10^. The presence of the macrolides narrows the diameter of the NPET, which reinforced the prevailing model that these drugs act as plugs for the tunnel and thereby inhibit translation of every protein indiscriminately (reviewed by ref. ^11^). However, this oversimplified view of macrolide action has been challenged in the past years with the finding that macrolides and ketolides are not global inhibitors of translation, but rather selectively interfere with the translation of a specific subset of proteins^12,13^.

Ribosome profiling analysis indicates that macrolides and ketolides can arrest translation at specific sequence signatures within the nascent polypeptide chain^14,15^. Interestingly, the specificity of action was shown to depend on the chemical structure of the macrolide, such that Arg/Lys-X-Arg/Lys, or so called “+X+” motifs (where + represent amino acids with positive charges and X is any amino acid) account for almost 80% of the strongest arrest sites in the presence of the ketolide TEL^14^. While macrolides, like ERY, also arrest translation at +X+ motifs, a more diverse range of arrest motifs, such as X+P, XDK, and XPW is observed^14–16^. In the macrolide-stalled ribosome, the arrest motif is located at the peptidyl-transferase center (PTC) and thus the macrolides inhibit translation by preventing the ribosome from catalyzing peptide bond formation^4,13,14^. For an Arg-Leu-Arg (+X+) motif, the ribosome stalls because the peptidyl-Arg-Leu-tRNA located at the P-site cannot undergo peptide bond formation with the incoming Arg-tRNA at the A-site^4,13,14,17,18^. For the +X+ motif, it appears that in addition to the positive charge of the Arg and Lys, the length of the side chain may play a role^18^, which may explain why Arg-X-Arg motifs induce an overall stronger arrest than Lys-X-Lys^14^. Importantly, because the arrest motif is located at the PTC, it is perceived as unable to establish direct contact with the macrolide bound deeper within the NPET, suggesting that macrolides exert their inhibitory action via the nascent chain and/or allosterically via nucleotides of the 23S rRNA^4,13,17^.

Importantly, arrest motifs, such as the +X+ motif, are found in many regulatory short upstream open reading frames (leader uORFs) that play a critical role in regulating the expression of macrolide-resistance genes, including rRNA methyltransferases (Erms)^17,19,20^. Erms methylate the N6 of A2058 (*E. coli* numbering used throughout) of the 23S rRNA^21^, which reduces the affinity of macrolides for their binding site by precluding water-mediated interactions between the desosamine sugar of the macrolide and A2058^10^. While Erms represent one of the most important mechanisms of resistance to macrolide antibiotics, the methylation of A2058 also confers a fitness cost to the bacteria^22^, therefore, Erm expression is tightly regulated via macrolide-dependent translation arrest^19,21^. One well-characterized example is the ErmDL leader uORF that regulates expression of the ErmD methyltransferase^23–25^. In the absence of ERY, ribosomes translate ErmDL, but the expression of the downstream ErmD methyltransferase is disfavored, presumably because of the unfavorable mRNA secondary structure (Fig. 1c). By contrast, in the presence of ERY, ribosomes become stalled at the Arg-Leu-Arg (+X+) arrest motif located between residues 6–8 of ErmDL, which in turn triggers mRNA refolding thereby favoring expression of the ErmD methyltransferase (Fig. 1d).

Despite the importance of the +X+ motif for general macrolide and ketolide inhibition, as well as the critical role of the +X+ motif for inducible Erm expression, a structural and molecular basis for how the drug, the ribosome and the +X+ motif interplay to mediate translational arrest has been lacking. Here, we employ the ErmDL leader uORF as a model system to study translational arrest at +X+ motifs in the presence of the macrolide ERY and the ketolide TEL. Using a combination of toeprinting and inverse toeprinting assays, we demonstrate that an intact +X+ motif is critical for ErmDL stalling in the presence of TEL, whereas it is dispensable for ERY-mediated programmed translation arrest. A molecular basis for these findings is revealed by cryo-EM structures of translating ribosomes stalled on the Arg6-Leu7-Arg8 sequence of ErmDL in the presence of ERY or TEL. In these structures, the drugs promote a conformation of the ErmDL nascent chain such that the side chain of Arg6 extends directly into the A-site pocket, where it would sterically clash with an incoming Arg-tRNA. In comparison to TEL, the conformation of ErmDL is further compacted by the presence of cladinose of ERY, to such an extent that the PTC of the ribosome cannot adopt the induced conformation necessary for accommodation of the A-site tRNA, thus explaining why the +X+ motif is dispensable for translational arrest in the presence of ERY. Collectively, our findings provide not only structural insights into the mechanism by which macrolides and ketolides interplay with the nascent chain to promote translation arrest, but also illustrate how specific chemical features of these antibiotics contribute to differentially modulate the conformation of the nascent chain and dramatically alter the mechanism of inhibition.

## Results

### The +X+ arrest motif is critical for ErmDL stalling in the presence of TEL

**Fig. 2:**
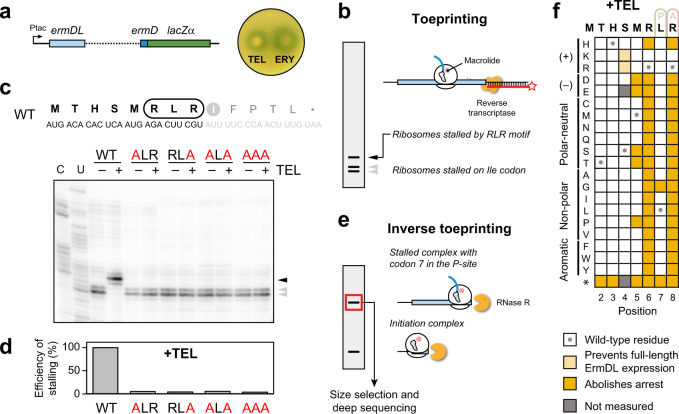
Sequence dependence of translation arrest at *ermDL* in response to TEL. **a** Induction of expression of the *ermD* reporter by macrolides. Left: schematic of the ermDL-ermD_1-6_-lacZα reporter construct. Right: Expression of the reporter, visualized as blue halos, is induced by diffusion of telithromycin (TEL) or erythromycin (ERY) on a lawn of *Escherichia coli* cells grown on LB agar reporter plates. **b** Overview of the toeprinting approach. Ribosomes that stall at a macrolide arrest motif (such as RLR) due to the presence of a macrolide antibiotic in a cell-free translation reaction produce a long cDNA band. Ribosomes that fail to stall at the arrest sequence are trapped at a downstream “hungry” codon (the trap codon) and produce a shorter cDNA band. **c** Top: The sequence of the *ermDL* ORF used for in vitro toeprinting assays. The RLR (Arg_6_-Leu_7_-Arg_8_) motif encoded in the ORF is highlighted. The original *ermDL* ORF was modified to contain the trap codon Ile-AUU (circled in gray) at position 9. Bottom: Toeprinting assay testing TEL-induced translation arrest at *ermDL* sequences, where the RLR motif is intact (wt) or contains the indicated Ala mutations. The toeprint band produced by TEL stalled ribosomes with the Leu7 codon at the P site is indicated by a black arrow. The toeprint bands of ribosomes stalled at codon 8 due to the lack of Ile-tRNA (due to the presence of the Ile-tRNA synthetase inhibitor mupirocin in the reactions), are shown with gray arrows. C-specific and U-specific sequencing reactions are shown. **d** Efficiency of TEL-mediated ribosome stalling estimated by quantifying the relative intensities of the toeprint bands of codons 7 and 8 in the mutant templates relative to those in the wt template. Stalling efficiency at the wt template was set to 100%. Circles indicate the values of two independent experiments, whereas the bar represents the mean. **e** Overview of the inverse toeprinting methodology. **f** Sequence-function map for seven positions of the ErmDL peptide translated in the presence of TEL, where mutations that abolish arrest (score < −1.5) are shown in yellow, as described in Supplementary Fig. 1. The ribosomal P (green) and A (pink) sites are indicated above the wild-type ErmDL sequence. Variants in gray were not measured and cells marked with a circle correspond to the wild-type amino acid. Mutations S4K and S4R lead to the appearance of a (K/R)MR arrest motif at position 4 that prevents the synthesis of the full peptide and leads to spuriously low scores for these two variants.

Induction of *ermD* by ERY had been demonstrated previously^23–25^, but it has remained unknown whether the ketolide TEL also serves as an inducer. To address this, we constructed a reporter plasmid containing the intact *ermDL* gene, the entire 276-nucleotide *ermDL*-*ermD* intergenic region as well as the first six codons of *ermD* fused in frame with the *lacZα* reporter (Fig. 2a) and introduced it into *E. coli* cells. The blue halo observed in a drug-diffusion assay^26^ suggests that not only ERY, but also TEL can readily activate the expression of the inducible *ermD* (Fig. 2a), possibly due to its ability to mediate programmed translation arrest within the *ermDL*-coding sequence, as reported previously for ERY^17,23–25^. Indeed, toeprinting analysis (Fig. 2b) showed that in a cell-free translation system, TEL directs ribosome arrest during translation of the Arg6-Leu7-Arg8 motif of ErmDL, when the Leu7 and Arg8 codons of the *ermDL* ORF are positioned at the P- and A-sites, respectively, of the stalled ribosome (Fig. 2c, d). By contrast, TEL was unable to stall the ribosome when the Arg6 and Arg8 codons of the *ermDL* ORF were mutagenized individually or simultaneously to Ala codons, or when all codons of the Arg-Leu-Arg sequence were changed to Ala (Fig. 2c, d). These results demonstrate the critical importance of the integrity of the Arg6-Leu7-Arg8 motif for TEL-mediated ribosome stalling on *ermDL*.

To evaluate more precisely the impact of the +X+ motif on TEL-dependent stalling and to also explore the possible role of the preceding N-terminal segment of the nascent ErmDL peptide, we employed inverse toeprinting^16^ to perform a deep mutational scan of *ermDL*. Like ribosome profiling^27^, inverse toeprinting can determine, with codon resolution, the position of paused ribosomes on a library of mRNA transcripts. This in vitro technique exploits a highly processive 3′ to 5′ RNA exonuclease (RNase R), such that the leading ribosome on each transcript protects the entire mRNA upstream of the pause site from degradation (Fig. 2e and Supplementary Fig. 1a). Therefore, a priori knowledge of the transcript sequences is not required and custom libraries of any complexity can be used. Here, we translated an mRNA library encoding all possible single amino acid substitutions at each of the positions 2–8 of ErmDL in the absence or presence of TEL, and produced ribosome-protected inverse toeprints. We then computed the change in each variant’s frequency observed by deep sequencing upon addition of TEL, yielding scores for 132 of the 134 possible ErmDL variants (Supplementary Fig. 1b), which reflect their effect on ribosome stalling. These scores followed a bimodal distribution (Supplementary Fig. 1c), with all tested mutations either having a neutral (> −1.5) or detrimental (< −1.5) effect on TEL-dependent stalling (Fig. 2f and Supplementary Fig. 1b). As expected, the mutation of Arg at positions 6 or 8 of ErmDL to residues other than Lys dramatically reduced TEL-dependent stalling (Fig. 2f and Supplementary Fig. 1b). We also noted that the mutation of Met5 to Pro, to a negatively charged (Asp, Glu), or to small polar amino acids (Ser, Thr) also negatively impacted stalling (Fig. 2f and Supplementary Fig. 1b). Thus, the results of inverse toeprinting confirm that TEL-dependent stalling is entirely dependent on the +X+ motif, in accordance with the general trends of ketolide-dependent arrest observed in vivo^14^.

### Cryo-EM structure of ErmDL-TEL-stalled ribosome complex

**Fig. 3:**
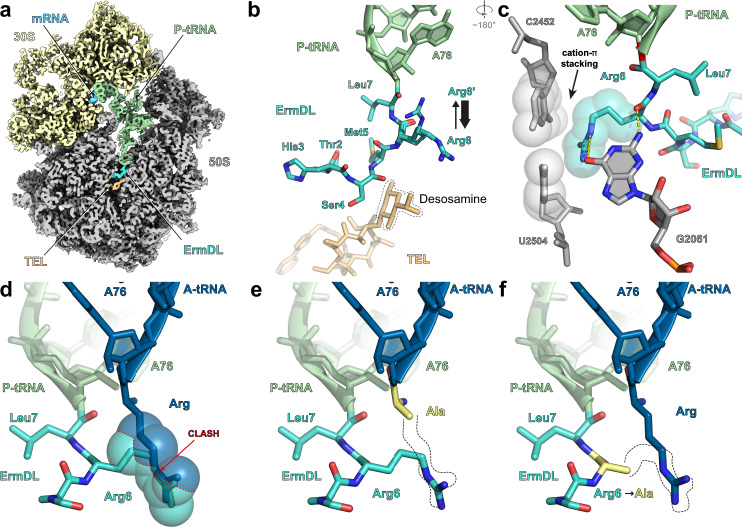
Cryo-EM structure of ErmDL-TEL-SRC. **a** Transverse section of the cryo-EM map of the ErmDL-TEL-SRC with isolated densities highlighting the 30S (yellow) and the 50S (gray) subunits, P-site tRNA (green), mRNA (gray), ErmDL nascent chain (cyan) and telithromycin (TEL, orange). **b** Molecular model of the ErmDL nascent chain (cyan), P-site tRNA (green) and telithromycin (TEL, orange) from the ErmDL-TEL-SRC. The desosamine sugar of TEL is highlighted with dashed line and the alternative conformations of Arg6 are shown with weighted arrows indicating relative stoichiometries based on density. **c** Interactions of Arg6 of ErmDL with 23S rRNA, including stacking interaction with the U2504-C2452 base pair (arrowed). Dashed lines to G2061 indicate potential hydrogen bonding with Arg6. **d** Superimposition of the ErmDL-TEL-SRC with an A-site Arg-tRNA in silico mutated from the ErmBL A-site Lys-tRNA (blue, PDB 5JTE)^28^, illustrating that Arg6 of ErmDL nascent chain clashes (indicated by spheres) with the Arg side chain of the accommodated A-site tRNA. **e**, **f** In silico mutations of **e** A-site tRNA to Ala (based on Phe-tRNA from pre-attack state PDB ID 1VY7)^31^, and **f** Arg6 to Ala mutation in ErmDL nascent chain.

To ascertain how TEL induces stalling at +X+ motifs, we determined a cryo-EM structure of an ErmDL-TEL-stalled ribosomal complex (SRC). To achieve this, an *E. coli* in vitro translation system was employed to translate a bicistronic *2XermDL* mRNA template in the presence of 20 μM TEL, similar to the approach described previously for generating ErmBL-SRCs and ErmCL-SRCs^28–30^. The resulting disomes of the ErmDL-TEL-SRC were isolated by sucrose gradient ultracentrifugation, converted to monosomes, and analyzed using single-particle cryo-EM (see “Methods” section). In silico sorting of the cryo-EM data revealed that the majority (76.4%) of ribosomes contained a P-site tRNA, but were heterogeneous with respect to presence or absence of A-site and/or E-site tRNAs (Supplementary Fig. 2a). We refined two subclasses, one containing only P-site tRNA (26.2%), and a second with stoichiometric A-site and P-site tRNAs (21.7%), yielding final reconstructions of the ErmDL-TEL-SRCs (Fig. 3) with average resolutions of 3.1 Å (Supplementary Fig. 2b) and local resolution extending towards 2.8 Å in the core of the large 50S subunit (Supplementary Fig. 2c, d). The density for the P-site tRNA was consistent with the presence of tRNA^Leu(GAG)^ base-pairing with the 7th codon (Leu CUU) of the *ermDL* mRNA (Supplementary Fig. 2e, f). Additional density was also observed for the variable region of tRNA^Leu(GAG)^ comprising a four G-C base pair stem and a four nucleotide (AAUA) loop (Supplementary Fig. 2e, f), which is shorter or absent in many other tRNAs, such as tRNA^fMet^ and tRNA^Phe^. Particularly well-resolved was the density for the CCA-end of the P-site tRNA, as well as for the ketolide TEL, the latter bound in an identical position to that observed previously in vacant ribosomes^7,9,10^ (Supplementary Fig. 2g, h). The density for the majority of the ErmDL nascent polypeptide chain was also well-resolved enabling Thr2 to Leu7 to be modeled de novo, except for Met1 that was apparently flexible precluding the N-terminal amino acid from being visualized (Fig. 3b and Supplementary Fig. 2g, h). The density for the side chain of Arg6 of ErmDL suggests that it adopts two alternative conformations, a minor one oriented back towards the A76 of the tRNA and a major one that stacks upon the U2504-C2452 base pair within the 23S rRNA (Fig. 3b, c and Supplementary Fig. 2h). The latter conformation of Arg6 of ErmDL is additionally stabilized by potential hydrogen bond interactions with the nucleobase of G2061 (Fig. 3c). We note that the density of the P-site tRNA, ErmDL, TEL and PTC nucleotides are identical (within the limits of the resolution) in the absence or presence of A-site tRNA. In the structure of the A-site tRNA-containing complex, the acceptor arm of the A-site tRNA was well-resolved and had accommodated on the 50S subunit, however, the CCA-end (and attached Arg residue) appeared to be flexible and no density was observed within the A-site pocket at the PTC.

Overall, the path of ErmDL in the presence of TEL is very different from that observed for other nascent polypeptide chains (Supplementary Fig. 3a–h), including the macrolide-dependent stallers ErmBL and ErmCL (Supplementary Fig. 3a–d)^28–30^. Rather than extending directly into the NPET as other nascent polypeptide chains (Supplementary Fig. 3a–h), ErmDL is oriented in a way that the C-terminal residues reach towards the A-site region then curl back past the desosamine sugar of TEL, such that the N-terminal residues can then extend into the lumen of the NPET (Fig. 3b). As a consequence of this conformation, in its predominant orientation (see above) the side chain for Arg6 extends directly into the A-site pocket at the PTC, where it would be predicted to clash with the arginyl (or lysinyl) moiety of an aminoacyl-tRNA (aa-tRNA) accommodated at the A-site (Fig. 3d and Supplementary Fig. 4a). By contrast, computationally substituting the Arg8 of the A-site tRNA with Ala (Fig. 3e) or mutating Arg6 to Ala (Fig. 3f) relieves the steric clash consistent with our functional assays demonstrating that, substitutions of Arg6 or Arg8 to Ala dramatically reduces the efficiency of ErmDL-mediated stalling in the presence of TEL (Fig. 2c, d,f). However, steric interference is apparently not sufficient to mediate stalling since superimposing an accommodated Phe-tRNA in the A-site from the pre-attack state^31^ also leads to clashes with Arg6 (Supplementary Fig. 4b), yet inverse toeprinting indicates that substitutions of Arg8 to bulky amino acids, including Phe, reduces the efficiency of stalling (Fig. 2f). Similarly, while substitutions of Arg6 of ErmDL to Lys would be predicted to produce clashes with Arg8-tRNA in the A-site (Supplementary Fig. 4c), consistent with the stalling observed by inverse toeprinting (Fig. 2f), we also note that Arg6 to Phe substitutions would also produce clashes with Arg8-tRNA in the A-site (Supplementary Fig. 4d), yet the inverse toeprinting results show that substitutions of Arg8 to bulky amino acids, including Phe, also reduces the efficiency of stalling (Fig. 2f). Thus, in addition to the size, it appears that the charge of the residue in the 6th and 8th positions of ErmDL may be important for stalling in the presence of TEL, as proposed in a previous study examining the macrolide-dependent stalling mechanism of a short MRLR peptide^18^ (see also Supplementary Notes and Supplementary Fig. 4e–h).

Our finding that the Arg-Leu-Arg (+X+) motif within ErmDL is critical for stalling in the presence of TEL is likely a manifestation of a more general phenomenon revealed by Ribo-seq data showing that ribosomes stall most often at Arg-X-Arg (+X+) motifs in the presence of TEL^14^. By contrast, while Ribo-seq data indicate that ribosome stalling also occurs at +X+ motifs in the presence of ERY^14,15^, the prevalence of stalling at +X+ motifs was much lower than for TEL and other sequence motifs were associated with the sites of ERY-induced arrest. These observations prompted us to assess the importance of the Arg-Leu-Arg (+X+) motif in ErmDL for ribosome stalling in the presence of ERY.

### The RLR motif is dispensable for ErmDL stalling in the presence of ERY

**Fig. 4:**
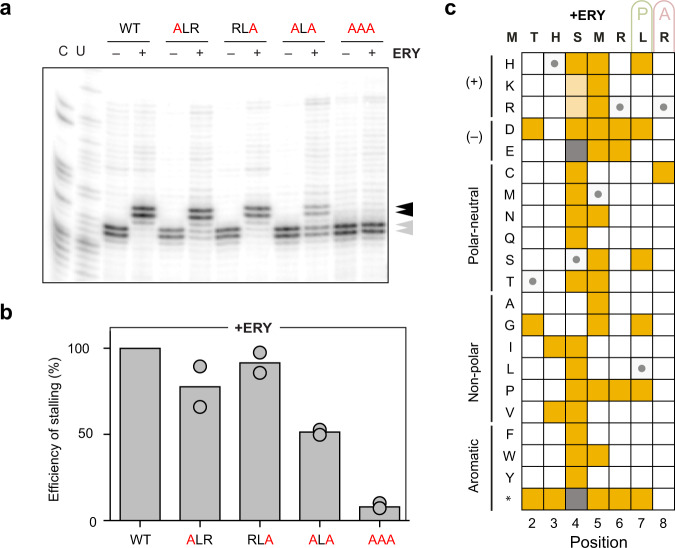
Sequence dependence of ErmDL arrest in the response to ERY. **a** The RLR motif of *ermDL* is not critical for ribosomes stalling with ERY. Toeprinting assay to assess translation arrest at *ermDL* sequences, with the original RLR motif (wt) or containing Ala mutations, in the presence of ERY. The toeprint bands of ERY stalled ribosomes at codon 7 (Leu or Ala) is indicated by a black arrow; the bands from ribosomes arrested at codon 8 because of the presence of mupirocin (see legend of Fig. 1d) are shown with gray arrows. Sequencing reaction lanes are labeled with C and U. **b** Efficiency of ribosome stalling with ERY estimated as described in the legend of Fig. 1d. Circles represent the values from two independent experiments, whereas the bar represents the mean value. **c** Sequence-function map for seven positions of the ErmDL peptide translated in the presence of ERY, where mutations that abolish arrest (score < −1.5) are shown in yellow, as described in Supplementary Fig. 1. The ribosomal P (green) and A (pink) sites are indicated above the wild-type ErmDL sequence. Variants in gray were not measured and cells marked with a circle correspond to the wild-type amino acid. Mutations S4K and S4R lead to the appearance of a (K/R)MR arrest motif at position 4 that prevents the synthesis of the full peptide and leads to spuriously low scores for these two variants.

Similar to TEL and consistent with previous reports^23–25^, ERY readily activates the expression of the *ermDL*-*ermD*_*1*−6_-*lacZ*α reporter in *E. coli* cells (Fig. 2a). Also in line with previously published data^17^, ERY stalls the ribosome at the Arg6-Leu7-Arg8 motif of *ermDL* during in vitro translation (Fig. 4a, b). However, in stark contrast with the TEL-induced translation arrest (Fig. 2c, d, f and Supplementary Fig. 1b), the integrity of the +X+ motif is less critical for ERY-dependent ribosome stalling, since Ala mutations of either Arg6 or Arg8 of *ermDL* only minimally affected stalling (Fig. 4a, b). Even the simultaneous Ala mutations of both Arg6 and 8 resulted in a fairly efficient arrest (nearly 50% of the stalling efficiency observed with *ermDL* encoding the original Arg6-Leu7-Arg8 motif), and only the consecutive mutation of the Arg6-Leu7-Arg8 triplet to Ala residues abolished ribosome stalling at *ermDL* with ERY (Fig. 4a, b). These results suggest that, in contrast with the requirements for TEL-dependent stalling, where the presence of the Arg6-Leu7-Arg8 motif plays a dominant role, the N-terminus of the ErmDL nascent chain also contributes to ERY-mediated programmed translation arrest within the *ermDL* ORF.

To expand upon this surprising finding, we employed inverse toeprinting and performed a deep mutational scan of *ermDL*, monitoring its translation in the absence or presence of ERY (Fig. 4c and Supplementary Fig. 1d). The inverse toeprinting data show that, in striking contrast to the critical role of the Arg residues at position 6 or 8 for TEL-dependent stalling, these same positions can be mutated to almost any other amino acid without compromising the ability of ErmDL to undergo ERY-dependent arrest (compare Figs. 2f and 4c). Notable exceptions are replacements of Arg6 with Asp, Glu, or Pro, as well as Arg8 with Cys, which reduce the efficiency of ERY-mediated arrest. Inverse toeprinting revealed that many single amino acid substitutions at positions 4 and 5 dramatically reduce ERY-dependent stalling, especially at position 4, where all substitutions of the Ser4 residue led to reduced stalling with the exception of Ala or Gly (Fig. 4c). Altogether, our inverse toeprinting data show that the N-terminal segment of ErmDL preceding the Arg6-Leu7-Arg8 sequence is sufficient to direct ERY-dependent stalling, and that ribosome arrest induced by ERY at the *ermDL* ORF does not rely exclusively on the +X+ motif. Thus, two closely related but distinct antibiotic molecules direct ribosome stalling with ErmDL by what appears to be two principally different mechanisms.

### Cryo-EM structure of ErmDL-ERY-SRC

**Fig. 5:**
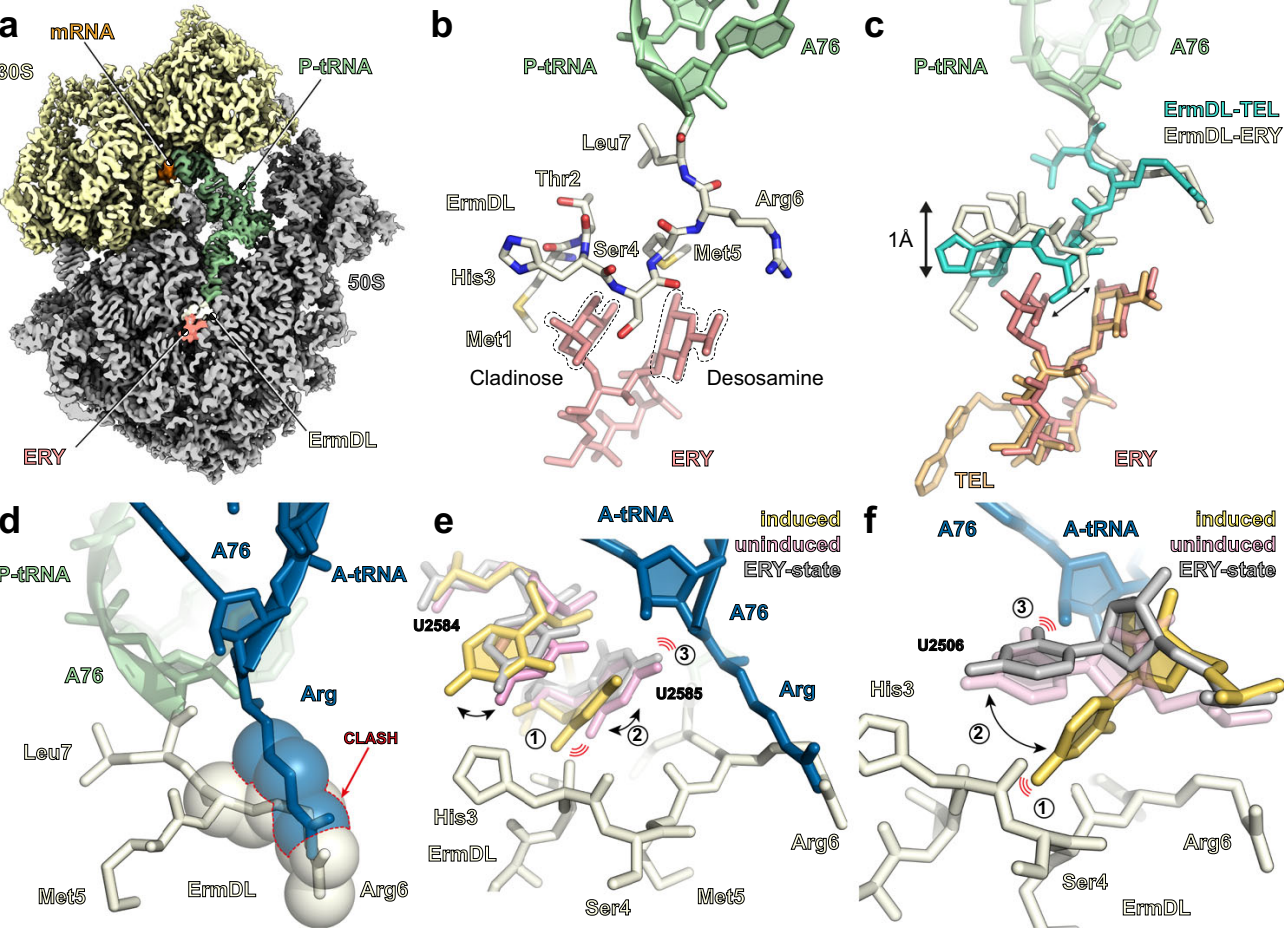
Cryo-EM structure of ErmDL-ERY-SRC. **a** Transverse section of the cryo-EM map of the ErmDL-ERY-SRC with isolated densities highlighting the 30S (yellow) and 50S (gray) subunits, P-site tRNA (green), mRNA (orange), ErmDL nascent chain (beige) and erythromycin (ERY, salmon). **b** Molecular model of the ErmDL nascent chain (beige), P-site tRNA (green) and erythromycin (ERY, salmon). The cladinose and desosamine sugars of ERY are highlighted with dashed line. **c** Superimposition of ErmDL nascent chains from the ErmDL-ERY-SRC (beige) and ErmDL-TEL-SRC (turquoise). Erythromycin (ERY) and telithromycin (TEL) from the respective structures are shown in salmon and orange. **d** Superimposition of the ErmDL-ERY-SRC with an A-site Arg-tRNA in silico mutated from the ErmBL A-site Lys-tRNA (blue, PDB 5JTE)^28^, illustrating that Arg6 of ErmDL nascent chain clashes with the Arg8 side chain of the accommodated A-site tRNA. **e**, **f** ErmDL stabilizes the uninduced state of the PTC to inhibit A-tRNA accommodation, as evident by comparison of the conformation of **e** U2584 and U2585 and **f** U2506 in ErmDL-ERY-SRC (gray) with the uninduced (pink, PDB 1VQN) and induced states (yellow, PDB 1VQ6)^34^.

To understand why stalling of ErmDL by ERY is distinct from that by TEL, we set out to determine a structure of an ErmDL-ERY-SRC. To do this, ErmDL-ERY-SRCs were generated as for the ErmDL-TEL-SRCs, except that TEL was substituted with ERY (see “Methods” section). In silico sorting of the cryo-EM data revealed that the majority of ribosomes contained a P-site tRNA (72%), with approximately half of the particles bearing an additional E-site tRNA (Supplementary Fig. 5a). Refinement of all the P-site tRNA containing particles yielded a final reconstruction of the ErmDL-ERY-SRC (Fig. 5a) with an average resolution of 2.9 Å (Supplementary Fig. 5b), and local resolution extending towards 2.5 Å in the core of the large 50S subunit (Supplementary Fig. 5c, d). As for the ErmDL-TEL-SRC, the density for the P-site tRNA was consistent with stalling occurring with tRNA^Leu(GAG)^ base-pairing with the 7th codon (Leu CUU) of the ErmDL mRNA (Supplementary Fig. 5e, f). Both the CCA-end of the P-site tRNA as well as the attached ErmDL nascent polypeptide chain were well-resolved, enabling all seven residues of ErmDL to be modeled unambiguously (Fig. 5b and Supplementary Fig. 5g, h). As expected, density was also observed for ERY bound in an identical position to that observed previously in vacant ribosomes^7,8^ as well as in ErmBL-SRCs and ErmCL-SRCs^28–30^. The density for the cladinose and desosamine sugars as well as the associated region of the macrolactone ring (C1–C6) of ERY was very well-resolved (Supplementary Fig. 5g, h), whereas the rest of the lactone ring appeared flexible and was observable only at lower thresholds, as observed previously for ErmBL-stalled and ErmCL-stalled ribosomes in the presence of ERY^28–30^. By contrast, this region of TEL in the ErmDL-TEL-SRC was well-ordered (Supplementary Fig. 5g, h), as observed previously for TEL^9,10^, possibly due to interaction of the heterocyclic sidechain of TEL with U2609-A752 basepair. We could also refine a minor (11.4%) subpopulation of ribosomes that contained A-site and P-site tRNAs, yielding a final reconstruction with an average resolution of 3.6 Å (Supplementary Fig. 5a). The position and conformation of the P-site tRNA, ErmDL, ERY and PTC nucleotides were indistinguishable (within the limits of the resolution) with or without A-site tRNA. As we observed for the A-site tRNA in the ErmDL-TEL-SRC, the acceptor stem of the A-site tRNA was also accommodated in the presence of ERY, yet no density for the CCA-end or the attached amino acid (Arg8) was observed within the A-site pocket at the PTC. Instead, the CCA-end appeared to adopt an alternative conformation where A76 stacked upon A2602 of the 23S rRNA (Supplementary Fig. 6a–c), analogous to that observed previously when the A-site pocket at the PTC was occupied by the antibiotic hygromycin A (Supplementary Fig. 6d–g)^32^.

Overall, the conformation of ErmDL in the presence of ERY is reminiscent of that observed in the presence of TEL with Arg6 also extending into the A-site pocket of the PTC (Fig. 5b, c), where it sterically clashes with the arginyl-moiety of an A-site Arg-tRNA (Fig. 5d). This is consistent with Ribo-seq data showing that, like with TEL, Arg/Lys-X-Arg/Lys (+X+) motifs are enriched at the sites of ERY-induced ribosome stalling^14,15^. But how then to rationalize our observations that ErmDL stalling in the presence of TEL is strictly dependent on an intact +X+ motif, but not in the presence of ERY? One obvious difference between the ErmDL-TEL-SRC and ErmDL-ERY-SRC structures is that the presence of the cladinose in ERY forces the ErmDL nascent chain to shift slightly towards the PTC when compared with TEL (Fig. 5c). Inversely, the absence of the cladinose sugar in TEL enables the ErmDL peptide to adopt a somewhat more relaxed conformation occupying this vacant space, and thereby extending slightly deeper into the NPET (Fig. 5c). Although this shift of the nascent chain is relatively modest, i.e., in the order of 1 Å, it is enough to cause a number of critical conformational changes of 23S rRNA nucleotides within the NPET and PTC. Firstly, A2062 of the 23S rRNA undergoes a rotation of approximately 90° compared to its position in the ErmDL-TEL-SRC structure, which appears to be the consequence of the altered position and conformation of the side chain of Met5 of ErmDL-ERY-SRC (Supplementary Fig. 7a–d). A second, and seemingly more relevant, consequence of the more compacted conformation of ErmDL in the presence of ERY is the inability of U2506 and U2585 to adopt the induced state conformation required for proper accommodation of the aa-tRNAs at the A-site^31,33,34^. In the conformation of these residues observed in the ErmDL-ERY-SRC, they impede placement of the acceptor substrate at the PTC by sterically clashing with the ribose of the 3′ terminal A76 of the A-site tRNA (Fig. 5e, f). In the induced conformation of these residues required for peptide bond formation they would clash with the backbone of the ErmDL peptide between His3 and Ser4 (Fig. 5e, f). By contrast, in the ErmDL-TEL-SRC structure, the shift in position of the ErmDL peptide deeper into the NPET is sufficient to allow an unhindered transition of U2585 and U2506 into the induced conformation (Supplementary Fig. 7e, f), which nevertheless does not lead to peptide bond formation due to the invasion of the A-site by the Arg6 side chain. The ErmDL-ERY-SRC structure also provides a possible explanation for the importance of Ser4 of ErmDL observed by the inverse toeprinting assay (Fig. 4c). In the structure, the side chain of Ser4 is tucked within a small cleft between the cladinose and desosamine sugars of ERY (Fig. 5b), which would be compatible with side chains of amino acids smaller than Ser, such as Ala or Gly, but incompatible with almost all other amino acids bearing large side chains. Therefore, larger side chains presumably perturb the conformation of ErmDL required for the arrest, or alternatively promote peptidyl-tRNA drop-off even before the ribosome reaches the site of the programmed translation arrest within the *ermDL* ORF (Fig. 4c).

Taken together, our findings rationalize the dramatic effect of the cladinose arm of the macrolide antibiotic on ErmDL stalling, such that in the presence of ERY, the cladinose sugar causes compaction of the ErmDL nascent chain that in turn prevents the PTC from adopting the induced conformation required for A-site tRNA accommodation. Thus, ribosome stalling still occurs in the presence of ERY despite those substitutions within the +X+ motif that remove steric clashes between the ErmDL peptide and the A-site tRNA. By contrast, in the presence of TEL, the ErmDL nascent chain is positioned slightly deeper in the NPET due to the absence of the cladinose sugar, providing the necessary space to allow the transition of the PTC into the induced state. This makes TEL-dependent stalling strictly dependent on the Arg6-Leu7-Arg8 (+X+) motif, which operates via invasion of the A site by the Arg6 side chain and thus prevents accommodation of the acceptor substrate (the Arg moiety of the Arg8-tRNA) into the A-site.

### Molecular dynamics simulations of ErmDL-SRCs

**Fig. 6:**
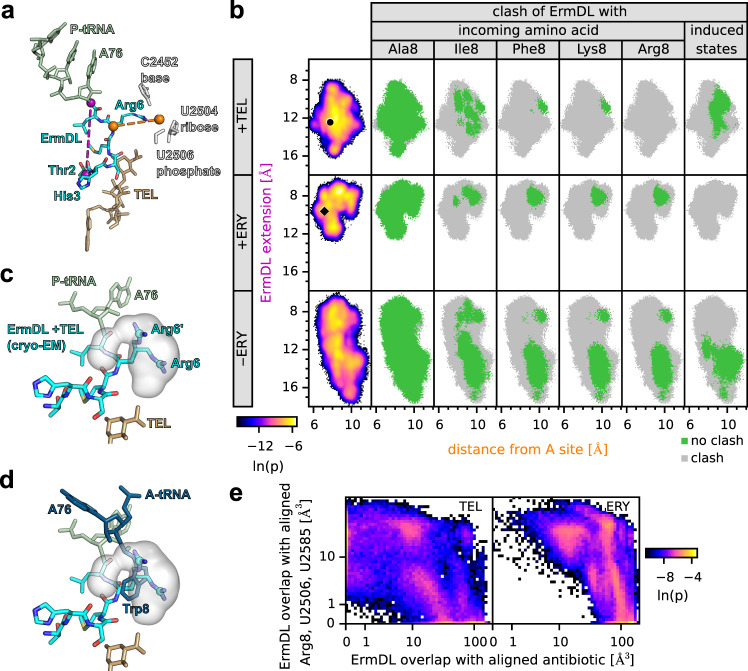
Dynamics of ErmDL obtained from MD simulations. **a** Molecular model of the ErmDL nascent chain, P-site tRNA, and telithromycin (TEL) from the ErmDL-TEL-SRC. The ErmDL extension (purple dashed line) is defined as the distance between O3′ atom of A76 (P-site tRNA) and Cα atom of Thr2. The position of the A site (right orange sphere) is defined as the center of geometry of the base-atoms, ribose-atoms, and phosphate-atoms of C2452, U2504, and U2506, respectively. The ErmDL distance from the A site (orange dashed line) is the distance between the Cα atom of Arg6 and the position of the A site. **b** Distance densities and clashes characterizing ErmDL fluctuations. The first column displays the logarithm (color coded) of the probability density *p* along the two distances indicated in **a**. The distances found in the cryo-EM structures with TEL and ERY (circle and diamond) are also shown. In columns 2–6, gray areas indicate distances where all conformations clash with the aligned Ala8, Ile8, Lys8, Arg8, and the induced states of 23S rRNA nucleotides (U2506, U2585), respectively. Green denotes regions with conformations that do not clash. **c**, **d** For the Cζ atom of Arg6, the probability density of its positions sampled in the +TEL simulations is shown (gray isosurface at p = exp(−10)) in comparison with the two Arg6 conformations (Cζ atoms shown as spheres) of the ErmDL-TEL-SRC cryo-EM structure (cyan). Comparison with rotamer conformation of Trp8 (**d**) that clashes with all conformations obtained from the +TEL MD simulations. **e** For simulations after the removal of ERY, the probability density of the van der Waals-overlap of ErmDL with the aligned antibiotic (TEL, left panel; ERY right panel) as well as with aligned A-site Arg8 and the induced nucleotides is shown.

To capture the effect of the antibiotics on the dynamics of the ErmDL nascent peptide, we performed all-atom explicit-solvent molecular dynamics (MD) simulations of ribosome-bound ErmDL in the presence or absence of ERY or TEL. The simulations involving TEL or ERY were started from the respective cryo-EM structures, whereas the simulations in the absence of antibiotic were initiated from the ERY-bound structure after computational removal of the drug. For each scenario (+TEL, +ERY, −ERY), we performed twenty 2-µs simulations encompassing all residues within a 35 Å distance of the P-tRNA CCA-end, the ErmDL peptide and ERY/TEL. To monitor the conformational dynamics of the ErmDL peptide, we measured two distances for each frame of each simulation, namely, the extension of the peptide (purple line in Fig. 6a) and the distance from the ErmDL Arg6 residue to the A site (orange line in Fig. 6a). Figure 6b (first column) shows the probability of each pair of distances calculated from all the simulations. In the presence of TEL, ErmDL explores conformations around that observed by cryo-EM (circle in Fig. 6b), extending between 8–16 Å and deviating by 6–11 Å from the A-site. By contrast, in the presence of ERY, the ErmDL peptide is more compact (7–13 Å) (Fig. 6b), reflecting the additional spatial restriction imposed by the cladinose sugar on ERY. Removing the drug (−ERY) promotes extension (7–18 Å) of the ErmDL peptide further down the NPET (Fig. 6b), where it occupies the space that has become available due to the absence of the drug (Supplementary Fig. 8a).

Arg6 of ErmDL in the presence of TEL is not restricted to a single conformation as indicated by the two conformations resolved by cryo-EM (Fig. 3b), which are also observed in the simulations (Fig. 6c). Therefore, we analyzed whether any of the explored Arg6 conformations alleviated the clash between Arg6 and different aa-tRNAs accommodated in the A-site of the PTC (Fig. 6b, columns 2–5 and Supplementary Fig. 8b). To that aim, we used the only available structures of ribosomal complexes containing A-site tRNAs, namely with Lys-tRNA^28^ and Phe-tRNA^31^, aligned all possible rotamers of the amino acids (Ala, Val, Ile, Phe, Met, Lys, Tyr, Trp, and Arg) to the Lys-tRNA and Phe-tRNA and subsequently selected the rotamers that did not overlap with rRNA nucleotides. For each frame of the simulation, we calculated van-der-Waals clashes of ErmDL with each of the selected rotamers. Most ErmDL conformations did not clash when the A-site tRNA was bearing small amino acids, such as Ala (Fig. 6b) and Val (Supplementary Fig. 8b), suggesting that these tRNAs can accommodate at the PTC and undergo peptide bond formation despite the presence of the side chain of Arg6 of ErmDL extending into the A-site. This is consistent with the inverse toeprinting analysis showing that mutation of Arg8 to small amino acids such as Ala or Val also relieved the translational stalling in the presence of TEL (Fig. 2f). As expected, we found that with increasing side chain length of the A-site amino acid, there was also an increase in the clashes observed with Arg6. While Ile displayed intermediate levels (Fig. 6b), for longer amino acids, such as Phe, Met, Tyr, all rotamers clashed with almost all Arg6 conformations (Fig. 6b and Supplementary Fig. 8b). This was particularly striking for Trp, where the single rotamer of Trp that is compatible with the A-site clashed with all conformations of Arg6 (Fig. 6d and Supplementary Fig. 8b). Since mutation of Arg8 to Trp relieved ErmDL stalling in the presence of TEL (Fig. 2f), this observation suggests that Trp (and most likely also Phe, Met, and Tyr) may displace Arg6 of ErmDL during A-site accommodation to undergo peptide bond formation. Despite the similarity in clashing profiles of Lys and Arg compared to other long amino acids, such as Phe, Met, Tyr, and Trp, the strong stalling indicates that these amino acids cannot displace Arg6 of ErmDL during accommodation and that another characteristic of the A-site amino acid, such as charge, contributes to stalling efficiency.

In the presence of ERY, we always observed a few conformations of ErmDL that did not produce a clash between Arg6 and the aminoacyl moiety of the A-site tRNA, regardless of whether it was Ala, Ile, Lys or Arg (Fig. 6b). This is consistent with our findings that for ErmDL-mediated translational stalling in the presence of ERY, an intact +X+ motif is dispensable (Fig. 4a–c). To understand why Arg6 can move out of the A-site in the presence of ERY, but not in the presence of TEL, we aligned the non-clashing Arg6 conformations from the +ERY simulations with the backbone of Arg6 in the TEL simulations. This revealed that all of the aligned Arg6 side-chain conformations from the +ERY simulations clash with either G2505 or U2506 in the ErmDL-TEL-SRC, indicating that these non-clashing conformations of Arg6 are incompatible with the backbone conformation of ErmDL in the presence of TEL. The structure of the ErmDL-ERY-SRC indicated that the ErmDL peptide adopts a compacted conformation in the presence of ERY that precludes the PTC from transitioning to the induced state. Therefore, we also analyzed whether any of the conformations explored by ErmDL could alleviate the clash between the ErmDL peptide and the induced conformation of U2506 and U2585 at the PTC^31,33,34^. In the simulations performed in the presence of TEL, we observed a subset of conformations that allowed U2506 and U2585 to adopt the induced state, whereas no conformations of ErmDL were observed in the presence of ERY that were compatible with the induced state (Fig. 6b, sixth column). An explanation for this finding is that in the presence of TEL, the ErmDL peptide extends deeper into the NPET, thereby providing the space required at the PTC for U2506 and U2585 to adopt the induced state (Supplementary Fig. 8c). By contrast, in the presence of ERY, the cladinose sugar of ERY prevents ErmDL from extending deeper into the NPET with the consequence that ErmDL is compacted at the PTC in a manner that precludes U2506 and U2585 from reaching the induced state (Supplementary Fig. 8d). This is corroborated by the simulations performed in the absence of drug (−ERY), where upon computational removal of ERY, the ErmDL peptide becomes much more flexible and extends deeper into the NPET, which by vacating the PTC provides multiple opportunities for U2506 and U2585 to attain the induced state (Fig. 6b and Supplementary Fig. 8a).

Crucially, this result also indicates that the timescales of the simulations are sufficient to observe conformational changes of ErmDL into non-stalling conformations in the absence of antibiotics, suggesting that it is indeed the antibiotics that hinder these conformational changes. To further explore whether the non-stalling conformations are sterically prevented by TEL and ERY for the simulations after removal of ERY, we first calculated the van-der-Waals overlap of the peptide with the region that would be occupied by Arg8 and the induced nucleotides. Second, to identify which conformations would be possible with TEL or ERY bound, we calculated the overlap with the region that would be occupied by either antibiotic. The probability density of these volumes shows that a large number of the ErmDL conformations would overlap with TEL and ERY (Fig. 6e). In particular, the conformations that we expect to alleviate stalling (no overlap with A-site Arg8 and induced nucleotides) only occur when the peptide has marked overlap with the antibiotic binding sites. These results underscore that the non-stalling conformations are generally inaccessible to ErmDL in the presence of ERY or TEL.

## Discussion

**Fig. 7:**
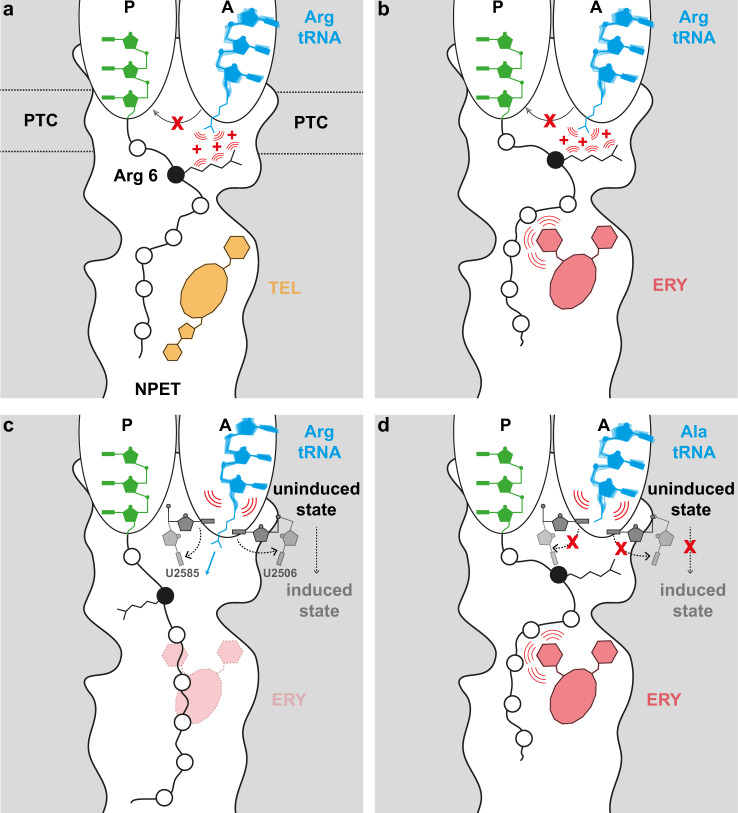
Model for the ErmDL-mediated translation arrest in presence of TEL or ERY. **a** The presence of TEL in the tunnel restricts the space available for the ErmDL nascent chain, constraining the ErmDL Arg6 side chain to extend into the A-site and thereby preventing accommodation of the incoming Arg-tRNA via charge repulsion. **b** The presence of ERY with its cladinose ring further restricts the space available for the ErmDL nascent chain constraining the ErmDL Arg6 side chain to extend into the A-site preventing via a charge repulsion mechanism the incoming Arg-tRNA in the A-site to adopt a stable conformation. **c** In the absence of ERY, the ErmDL nascent chain freely explores the NPET and adopts an extended conformation. During the accommodation of an incoming A-tRNA, 23S residues U2506 and U2585 shift from their uninduced state to their induced state. **d** In the presence of ERY, the compacted conformation of ErmDL prevents the PTC from adopting the induced conformation necessary for accommodation of the A-site Ala-tRNA independently of the charge repulsion mechanism from **c**.

Here we have employed the ErmDL leader uORF that bears an internal Arg6-Leu7-Arg8 (+X+) motif as a model to investigate the molecular basis of translation arrest at these sequences by the macrolide ERY and the ketolide TEL. We have demonstrated that in the presence of TEL, an intact +X+ motif is critical since substitution of Arg6 or Arg8 to any other amino acid, except Lys, abolished the translational arrest (Fig. 2b, c, f). A structure of the ErmDL-TEL-SRC revealed that, in the presence of TEL, the ErmDL nascent chain adopts a defined conformation in which Arg6 of ErmDL is directed into the A-site of the PTC active site, where it would clash with the incoming Arg8, thus providing a simple explanation for how TEL and ErmDL interplay to arrest translation. Consistently, mutation of Arg8 to Ala alleviates the steric clash (Fig. 3e) and accordingly relieves the translational arrest (Fig. 2b, c, f). The extent of clashing between the penultimate residue of the ErmDL nascent chain and the incoming amino acid plays an important role; indeed, Arg and Lys are the residues with the longest side chains among the proteinogenic amino acids. However, our biochemical data and MD simulations suggest that length of the side chains at these positions is not the only factor for stalling because replacement of Arg6 or Arg8 with comparably bulky amino acids, is not predicted to resolve the clash, but nevertheless prevents TEL-induced translation arrest (Fig. 2f). This is highlighted by the mutation of Arg8 to Trp, which alleviates the stalling (Fig. 2f), yet is not predicted to resolve the clash with Arg6 (Supplementary Fig. 8b). Since the relief of stalling requires the accommodation of the Trp-tRNA at the A-site so that peptide bond formation can ensue, we assume that in the case of amino acids with large side chains (other than Lys or Arg), the incoming acceptor substrate can displace Arg6 of ErmDL from the A-site of the PTC. This raises the question as to why other amino acids can achieve this displacement, whereas Lys and Arg cannot? A likely answer is that in addition to carrying the longest side chains, Lys and Arg are also unique in that they are both positively charged so that electrostatic repulsion helps to prevent the Lys8-tRNA or Arg8-tRNA accommodation (Fig. 7a), as previously proposed for macrolide-dependent stalling of short MRLR sequences^18^. Furthermore, the positive charge at the end of the ErmDL’s penultimate amino acid could be a prerequisite for the A site invasion due to its stacking interaction with the U2504-C2452 base pair (Fig. 3c). We note that in the ErmDL-TEL-SRC, we observed two alternative conformations for Arg6 (Fig. 3b) and multiple conformations in the simulations that may contribute to the repulsion of the incoming positively charged aminoacyl-tRNA (Fig. 6c). Finally, the mutation of Arg6 to any other amino acid except for the positively charged Lys, also removes the repulsion effect, thus allowing accommodation and peptide bond formation and thereby relieving the translational stalling, re-emphasizing the critical role of the +X+ motif in the TEL-induced translation arrest of ErmDL. Because we observed a similar conformation of ErmDL in the presence of ERY, where the side chain of Arg6 extends into the A-site, it is likely that in most cases, stalling at +X+ motifs in the presence of ERY may also utilize a charge repulsion mechanism, analogous to that outlined above for TEL (Fig. 7b). In our MD simulations, removal of the antibiotic enabled the nascent chain to extend deeper into the ribosomal tunnel, leading to the retraction of the side chain of Arg6 from the A-site pocket at the PTC (Fig. 7c). This explains why translational stalling at +X+ motifs is not observed in the absence of the drugs^14,15^.

It is noteworthy that in the presence of TEL or ERY, ribosomes do not stall at every +X+ motif present within proteins^14,15^, indicating that the context of the +X+ motif also plays an important role (Supplementary Fig. 9a). In keeping with this notion, changing the ErmDL N-terminal sequence from MTHSMRLR to MDTLNRLR by compensatory frame-shifting mutations alleviates translation arrest at the Arg6-Leu7-Arg8 (+X+) sequence of the modified ORF with either ERY or TEL (Supplementary Fig. 9b). In light of our ErmDL-SRC structures, one can envisage that some amino acid sequences N-terminal to the +X+ motif, juxtaposed against the drug molecule in the NPET, could alter the conformation of the nascent chain such that, even in the presence of the macrolide/ketolide, the side chain of the first positively charged Lys/Arg residues of the +X+ motif does not extend into the A-site, thereby allowing accommodation of the second Lys/Arg-tRNA at the A-site leading to productive peptide bond formation.

Our study has revealed that both TEL and ERY arrest the ribosome, when it reaches the 7th codon of the *ermDL* uORF. Given the overall structural similarity of the two related drugs, it is striking that they utilize seemingly principally different mechanisms for ribosome stalling. Given the conservation of +X+ motifs in the regulatory uORFs of macrolide resistance genes (Sothiselvam et al.^17^), it was counterintuitive that the +X+ motif of ErmDL plays little role in the arrest of the ribosome prompted by the presence of ERY (Fig. 4a–c). Instead, the residues more proximal to the ErmDL N-terminus and more remote from the site of arrest at Leu7, such as Ser4 and Met5, were found to be critical for ERY-dependent stalling (Fig. 4c). The structure of the ERY-ErmDL-SRC provides a likely explanation of why ERY-dependent stalling does not rely on the presence of the +X+ motif. During processive translation, aminoacyl-tRNAs accommodate at the A-site of the PTC through an induced fit mechanism requiring the shift of nucleotides U2506 and U2585 from uninduced to induced conformations (Fig. 7c). By contrast, the presence of ERY in the NPET causes compaction of the N-terminal segment of the ErmDL peptide, such that the PTC cannot adopt the conformation necessary for productive accommodation of the A-site tRNA (Fig. 7d). A comparison of the ErmDL-TEL-SRC and ErmDL-ERY-SRC structures shows that it is the C5 cladinose present in ERY, but absent in TEL, that is responsible for the ErmDL compaction. Such compaction depends on the structure of the nascent chain N-terminal to the site of arrest, as revealed both by the results of deep single amino acid mutagenesis (Fig. 4c), and by frameshifting of the *ermDL* N-terminal coding sequence (Supplementary Fig. 9c). The dependence of ERY-dependent translation arrest on other determinants also explains, why ribosome stalling in the presence of TEL most commonly calls for the presence of the +X+ motif, whereas more diverse motifs are observed at the sites of the ERY-induced arrest in the bacterial genomes as well as the sites of arrest induced by a 15-membered macrolide azithromycin that also bears a C5 cladinose^14,15^.

Our finding that TEL-and ERY-dependent ribosome stalling at the regulatory ORF of the *ermD* resistance gene operates through different mechanisms begs the question of the origin of such dichotomy. The resistance genes are often found in the producers of natural antibiotics or in organisms that are exposed to such antibiotics in their natural environment. Noteworthy, the resistance in the producer of the natural ketolide pikromycin is activated exclusively by ketolides^20^, whereas some macrolide resistance genes are selectively induced by C3 cladinose-containing macrolides^35^. While the origin of the *ermD* gene and its regulatory circuit is unknown, it is possible that it first evolved in the producer of a macrolide antibiotic, and that its induction relied on the compaction of the N-terminal segment of the leader peptide in the macrolide-obstructed NPET. Subsequent acquisition of the ketolide biosynthesis gene cluster could then select for the RLR sequence that would allow induction of the resistance by both types of antibiotics. Alternatively, exposure of bacteria to cladinose-containing macrolides in the environment could lead to selection or acquisition of a macrolide-inducible resistance gene, with subsequent exposure to natural ketolides forcing selection of the +X+ motif in the leader peptide. Importantly, the latter scenario illustrates how the resistance to ketolides could easily evolve in the clinic in pathogens that carry inducible resistance genes originally responding exclusively to macrolides.

Finally, we note that the mechanisms identified here for ErmDL are quite distinct from those observed previously for other macrolide-dependent stalling peptides, for example, ErmBL^28,30^ and ErmCL^29^. In the ErmBL-ERY-SRC, accommodation of the A-site tRNA is observed at the PTC, however, peptide bond formation cannot occur because the interplay between ERY and ErmBL creates a suboptimal geometry of the P-site tRNA^28,30^. By contrast, in the ErmCL-ERY-SRC, the drug and ErmCL peptide induce conformational changes within the PTC, in particular rotation of U2585, such that accommodation of the A-site tRNA is prohibited. It will be interesting in the future to investigate whether distinct mechanisms are utilized by other drug-dependent nascent polypeptide chain stalling systems to inactivate the PTC of the ribosome and induce translational arrest.

## Methods

### Reagents

Mupirocin and ERY were from Sigma Aldrich, telithromycin was from Cempra, Inc. All other reagents were from ThermoFisher. DNA oligonucleotides were synthesized by Integrated DNA Technologies.

### Construction of the inducible *ermD* reporter and antibiotic diffusion test

A segment of the *ermDL-ermD* operon (encompassing the *ermDL* coding region, the intergenic segment and the first six codons of the *ermD* gene) was amplified from plasmid pBD244 found in the *Bacillus subtilis* strain BG28^24^ and kindly provided by Dr. Bechhofer (Mount Sinai School of Medicine, NY). The PCR reaction was carried out using the primers ermDL-NdeI-forward and ermD-AflII-reverse (Supplementary Table 1). The resulting PCR fragment was used to substitute the *ermCL-ermC* portion of pErmZ101t^36^ to yield the plasmid pErmZ101t-ermD. *E. coli* strain JM109 cells were transformed with the pErmZ101t-ermD plasmid and the induction of the ermD_1-6_-lacZα reporter by antibiotics was tested by the diffusion assay as previously described^26^, with minor modifications. Specifically, cells were grown to an A_600_ of 1.0 and then 0.5 mL of the culture were mixed with 3.5 mL of soft agar [0.6% (wt/vol) LB agar] at 50 °C and overlaid on agar plates containing 10 μg/mL tetracycline, 0.5 mM IPTG, 80 μg/mL X-Gal, and 0.25 mM of the β-galactosidase inhibitor phenylethyl-b-d-thiogalactopyranoside (PETG). A 1 µL drop of a solution containing 25 µg of ERY or 10 µg of TEL were placed on top of the solidified soft agar. Plates were incubated at 37 °C for 18–24 h and photographed.

### Toeprinting assay

Linear DNA templates encoding modified versions of the *ermDL* ORF, preceded by the T7 promoter sequence and including the toeprinting primer (NV1) annealing region, were prepared by PCR. Primers T7-MTHSM-fwd and NV1-IFPTL-rev (Supplementary Table 1) at 0.4 µM were mixed with 0.04 µM of forward and reverse primers (Supplementary Table 1) encoding the *ermDL*-derived ORFs with the original Arg_6_-Leu_7_-Arg_8_ stalling site (RLR-IFPTL fwd and RLR-IFPTL rev) or its mutant versions. An Ile codon (ATT) was introduced as codon 9 of the templates and codons Asn_13_ (ACC) and Gln_14_ (CAG) of the original *ermDL* were omitted. The template with no Ala-mutations of the Arg_6_-Leu_7_-Arg_8_ stalling motif is referred to as wt-*ermDL*. Templates for the WT *ermDL* and its frame-shifted (FS) mutant version were prepared by PCR combining primers WT-MTHSM-fwd or M3-MDTLN-fwd, respectively, with reverse primer ermD-58 nts-rev (Supplementary Table 1). AccuPrime DNA polymerase was used for all PCR reactions. Coupled transcription-translation reactions was carried out in 5 µL of the PURExpress system (New England Biolabs) containing additional 3 mM of MgCl_2_ followed by reverse transcriptase-mediated extension of the NV1 primer (Supplementary Table 1), as described previously^37^. Reactions were also supplemented with the Ile-RS inhibitor mupirocin (50 µM) and, where indicated, with erythromycin (50 µM) or telithromycin (100 µM). The cDNA extension products, along with sequencing reactions of the of the wt-*ermDL* template, were separated in 6% sequencing gels, dried, exposed, and visualized in a Typhoon scanner (GE Healthcare). Intensity of the toeprint bands was quantified using ImageJ^38^. Relative efficiency of antibiotic-mediated stalling was calculated using formula *E*_stalling_ = *I*_7_/*I*_7_ + *I*_8_, where *I*_7_ is the intensity of the toeprint band corresponding to the ribosome stalled with the 7th codon of the *ermDL* template in the P site, representing macrolide-dependent translation arrest, and *I*_8_ is the intensity of the toeprint band at the 8th codon, representing macrolide-independent translation arrest.

### Deep mutational scan of ErmDL

#### Single-codon ermDL variant library generation

A library of single-codon *ermDL* variants was generated by mixing equimolar amounts of linear double stranded DNA expression cassettes in which codons 2–8 of *ermDL* had individually been replaced with an “NNS” codon (aNy aNy Strong (G/C)). These expression cassettes were first obtained by polymerase chain reaction (PCR) with Phusion DNA polymerase (20 cycles [98 °C, 10 s; 60 °C, 5 s; and 72 °C, 10 s]), using one of seven degenerate oligonucleotides encoding *ermDL* (ermDL-NNS2 to 8) in combination with oligonucleotides T7_RBS_ATG_f and Stop_EcoRV_r as templates (1 pmol of each oligonucleotide per 50 μL reaction), and oligonucleotides T7_f and EcoRV_r for amplification (10 pmol of each oligonucleotide per 50 μL reaction). The sequence of the expression cassette for wild-type *ermDL* is CGATCGAATTCTAATACGACTCACTATAGGGCTTAAGTATAAGGAGGAAAAAATATG**ACACACTCAATGAGACTTCGT**GCGATCTCGGTGTGATGA*GATATC*AATATCAAAAAGGATCCATATA (the T7 promoter is underlined, the mutated *ermDL* region is in **bold**; the EcoRV site is in *italics*). This linear expression cassette was purified using a PCR purification kit (Qiagen) according to the manufacturer’s instructions and quantified using a 2100 Agilent Bioanalyzer.

#### Inverse toeprinting

In vitro transcription was performed at 20 °C for 3 h, using T7 RNA polymerase in a buffer containing 80 mM Tris-HCl, 24 mM MgCl2, 2 mM spermidine, and 40 mM DTT, pH 7.6, in the presence of 7.5 mM ATP (Sigma Aldrich), CTP and UTP, 0.3 mM GTP (CTP, UTP, and GTP from Jena Bioscience), and 4.5 mM Thio-Phosphate-GMP (Genaxxon Bioscience). Eight picomoles of DNA template were used in a 200 μL reaction volume. Transcripts were purified using the “RNA Clean & Concentrator^TM^−5” purification kit (ZymoClean Research) according to the manufacturer’s instructions. The resulting mRNA library was biotinylated and polyadenylated as described previously^16^.

In vitro translation reactions (5 μL) were carried out with a PURExpress ΔRF-123 ΔRibosome kit (NEB), using ~5 pmol of 5′-biotinylated and 3′-polyadenylated mRNA as a template. Antibiotics (ERY, TEL) were added to a final concentration of 50 μM. Release factors 1 and 3 were added to the translation reaction according to the manufacturer’s instructions. Translation was performed at 37 °C for 30 min, after which the samples were placed on ice and 5 μL ice-cold Mg^2+^ buffer (50 mM Hepes-KOH pH 7.5, 100 mM K-glutamate, 87 mM Mg-acetate, 1 mM DTT) was added to the reactions, thereby increasing the Mg^2+^ concentration to 50 mM. One microliter of RNase R (1 mg/mL) was added, followed by additional incubation for 30 min at 37 °C to ensure complete degradation of the mRNA. One hundred and thirty-nine microliter of 1× BWT buffer was added to stop the reaction (5 mM Tris-HCl, 0.5 mM EDTA, 1 M NaCl, and 0.05% [vol/vol] Tween- 20, pH 7.5).

For each sample, 5 μL of M-280 streptavidin Dynabeads (Thermo Fisher Scientific) washed and then diluted in 50 μL of 1× BWT buffer were added to all of the digested RNA, and the mixture was incubated for 15 min at room temperature. After incubation, beads were washed once with 1× BWT buffer to remove unbound RNA, followed by two washes with water to remove the 1× BWT buffer. Beads were resuspended in 9.5 μL of linker ligation reaction mixture containing 4 μL of water, 1 μL of T4 RNA ligase2 truncated buffer (10×—NEB), 3 μL of PEG 8000 (50%—NEB), 1 μL of 3′_linker_ApoI (10 μM) and 0.5 μL of ligase (T4 RNA ligase 2, truncated—200,000 U/mL—NEB) per reaction. Linker ligation was allowed to proceed for 2.5 h at room temperature. Beads were washed once with water to remove unincorporated linker oligonucleotide and resuspended in 18.5 μL of reverse transcription reaction mixture containing 11.5 μL of water, 1 μL of dNTPs (10 mM of each - NEB), 1 μL of Linker_r oligonucleotide (2 μM), 4 μL of first strand synthesis buffer (5×—Thermo Fisher Scientific) and 1 μL of DTT (0.1 M—Thermo Fisher Scientific) per reaction. Samples were incubated for 5 min at 65 °C before adding 1 μL of reverse transcriptase (Superscript III—200,000 U/mL—Thermo Fisher Scientific) to each tube and incubating for 30 min at 55 °C.

To generate double stranded DNA for restriction digestion, a fill-up reaction was performed using 10 pmol of cDNA_f oligonucleotide and all of the reverse transcribed cDNA (10 s denaturation, 10 s annealing at 42 °C, and 30 s elongation at 72 °C). The resulting double stranded DNA was combined with 1 μL of EcoRV restriction enzyme and the sample was incubated at 37 °C for 1 h. Undigested DNA was mixed with 10 pmol of Linker_r oligonucleotide and amplified by 10–16 cycles of PCR [98 °C, 10 s; 60 °C, 10 s; and 72 °C, 10 s]. The number of PCR cycles was adjusted to give a visible band on the gel while minimizing non-specific byproducts. Bands containing inverse toeprints corresponding to stalled ribosomes with codon 7 of *ermDL* in P-site were excised from the gel with a clean scalpel. Gel pieces were crushed through a 5 mL syringe into tubes containing 5 mL of gel elution buffer (10 mM Tris-HCl, pH 8.0, 500 mM Na-acetate, and 0.5 mM Na-EDTA), and DNA was extracted overnight at room temperature. After removal of the gel debris, each sample was concentrated to ~1 mL using a SpeedVac. DNA was precipitated with 1 mL of isopropanol in the presence of 3.7 μL GlycoBlue (Thermo Fisher Scientific), recovered by centrifugation at 20,000×*g* for 30 min at 4 °C and resuspended in 20 μL water.

#### Next generation sequencing of inverse toeprints

Long NGS adapter oligonucleotides (NGS_adaptor_f and the reverse oligonucleotides NGS_adaptor_23 through NGS_adaptor_23 + 3) contain Illumina TruSeq adapter sequences followed by 18 nucleotides complementary to the 5′ or 3′ region of the cDNA. The reverse oligonucleotides also contain barcode sequences for multiplexing according to the TruSeq v1/v2/LT protocol (Illumina). Processed inverse toeprints were amplified by 12–16 cycles of PCR, using 0.02 μM long NGS adapter oligonucleotides (forward and reverse) and 0.2 μM short amplification oligonucleotides (NGS_f and NGS_r). The resulting NGS libraries were purified using a Qiagen PCR purification kit. The size and concentration of the fragments thus obtained were determined using a 2100 Agilent Bioanalyzer with the DNA 1000 kit. Next generation sequencing was performed by the BGI Facility in Hong-Kong, on an Illumina HiseqXten system in rapid run mode with 150 PE reads.

#### Analysis of ErmDL deep mutational scanning data

Unless it is indicated otherwise, data analysis was carried out using a series of custom scripts written in-house in Python, which relied on the use of the Biopython package^39^. Read pairs were assembled using PEAR v0.9.10^40^ on a Mac Book Pro with a 2.7 GHz Intel Core i7 processor and 16 GB 1600 MHz DDR3 memory with the maximal proportion of uncalled bases in a read set to 0 (–u option), and the upper bound for the resulting quality score set to 126 (–c option). Regions immediately upstream of the start codon and downstream of the point of cleavage by RNase R were removed using a modified version of the *adaptor_trim.py* script written by Brad Chapman (https://github.com/chapmanb/bcbb/blob/master/align/adaptor_trim.py). The 5′ flanking region was defined as GTATAAGGAGGAAAAAAT, whereas the 3′ flanking region was GGTATCTCGGTGTGACTG. A maximum of two mismatches within each of these flanking regions was tolerated, whereas all other reads were discarded. Trimming of the retained reads resulted in sequences with a start codon directly at the 5′ end and the site of RNase R cleavage at the 3′ end. Trimmed reads were analyzed in Enrich2 v1.0.0^41^ in a single run with two separate experimental conditions (ERY, TEL), each consisting of 6 replicates (2 biological × 3 technical) of the libraries obtained following selection in the absence or presence or drug. Reads were required to have a minimum quality score of 30 at all positions and contain no Ns.

### Sample and grid preparation

The ErmDL construct and ribosome complexes were generated following the same procedure as previously described^28–30^. Briefly, under the control of the T7 promoter, two consecutive ErmDL ORFs with strong ribosome-binding site upstream of the ATG start codon were synthesized (Eurofins, Germany). Both ErmDL ORFs were separated by 22 nt linker enabling an independent initiation of both ORFs and later on the hybridization of complementary DNA oligonucleotide required for RNase H cleavage. In vitro coupled transcription and translation of the 2× ErmDL construct was performed using the Rapid Translation System RTS 100 *E. coli* HY Kit (biotechrabbit GmbH, berlin) in presence of 10 μM ERY or 10 µM of TEL for 1 h at 37 °C. The ErmDL-stalled disomes were isolated on 10-40% sucrose gradients in buffer A, containing 25 mM HEPES-KOH pH 7.5, 150 mM KOAc, 15 mM Mg(OAc)_2_, 1 mM DTT, 0.01% N-dodecyl β-d-Maltoside, 50 μM erythromycin or telithromycin, by centrifugation at 34,380 × *g* (SW-40 Ti, Beckman Coulter) overnight at 4 °C. The disome fraction was then pelleted by centrifugation at 109,760 × *g* (Ti70.1, Beckman Coulter) overnight at 4 °C and re-suspended in buffer B, containing 25 mM HEPES-KOH pH 7.5, 100 mM KOAc, 25 mM Mg(OAc)_2_. Conversion into monosomes was performed first by annealing of a complementary DNA oligonucleotide (5′-TTCCTCCTTATAAAACT-3′) for 5 min at 30 °C, directly followed by a cleavage of the RNA-DNA hybrid by RNase H (Thermo Fisher Scientific) at 25 °C for 1 h. The reaction was layered on 10–40% sucrose gradient in buffer A and the monosomes were fractionated by centrifugation at 34,380 × *g* (SW-40 Ti, Beckman Coulter), directly followed by a pelleting centrifugation step at 109,760 × *g* (Ti70.1, Beckman Coulter) overnight at 4 °C. The stalled monosomes were re-suspended in buffer B to a final concentration of 150 *A*_260nm_/mL ErmDL-TEL-SRC and 100 *A*_260nm_/mL ErmDL-ERY-SRC. For grid preparation, 4 μL (5 *A*_260_/mL) of the freshly prepared ErmDL-TEL-SRC or ErmDL-ERY-SRC complex was applied to 2 nm precoated Quantifoil R3/3 holey carbon supported grids and vitrified using a Vitrobot Mark IV (FEI, Netherlands).

### Cryo-electron microscopy and single-particle reconstruction

Data collection of both ErmDL-TEL-SRC and ErmDL-ERY-SRC was performed on a FEI Titan Krios transmission electron microscope (TEM) (Thermo Fisher) equipped with a Falcon II direct electron detector (FEI). Data were collected at 300 kV with a total dose of 25 e^−^/Å^2^ fractionated over 10 frames with a pixel size of 1.084 Å/pixel and a target defocus range of −0.7 to −3 μm using the EPU software (Thermo Fisher). The raw movie frames were summed and corrected for drift and beam-induced motion at the micrograph level using RELION-3.0^42^. The resolution range of each micrograph and the contrast transfer function (CTF) were estimated with Gctf^43^. For ErmDL-TEL-SRC, a total of 5850 micrographs were collected. After manual inspection, 5430 micrographs were used for automated particle picking with Gautomatch (http://www.mrc-lmb.cam.ac.uk/kzhang/) resulting in 433,827 initial particles, of which 384,895 were selected for further processing upon 2D classification in RELION-3.0^42^. After initial alignment with a vacant 70S reference, the 384,895 particles (defined as 100%) were 3D classified into eight classes (Supplementary Fig. 2a). Class 4 (83,696 particles, 21.7%) and class 8 (100,679 particles, 26.2%) were further selected for 3D and CTF refinement using RELION-3.0^42^. The final reconstructions were corrected for the modulation transfer function of the Falcon 2 detector and sharpened by applying a negative B-factor estimated by RELION-3.0^42^. The resolution for the class 4 and 8 ErmDL-TEL-SRC were estimated using the “gold standard” criterion (FSC = 0.143)^44^ resulting in a final reconstruction of 3.1 Å (Supplementary Fig. 2b). Local-resolution estimation and local filtering of the final volumes were done using Relion-3.0 (Supplementary Fig. 2c–e). For ErmDL-ERY-SRC, a total of 3850 micrographs were collected. After manual inspection, 3170 micrographs were used for automated particle picking with Gautomatch (http://www.mrc-lmb.cam.ac.uk/kzhang/) resulting in 288,013 initial particles, of which 240,530 were selected for further processing upon 2D classification in RELION-3.0^42^. After initial alignment with a vacant 70S reference, the 240,530 particles (defined as 100%) were 3D classified into eight classes (Supplementary Fig. 5a). Class 2 (27,397 particles, 11.4%) and class 3, 4, 7, and 8 (172,175 particles, 71.6%) were further selected for 3D and CTF refinement using RELION-3.0^42^. The final reconstructions were corrected for the modulation transfer function of the Falcon 2 detector, and sharpened by applying a negative B-factor estimated by RELION-3.0^42^. The resolution for the ErmDL-TEL-SRC was estimated using the “gold standard” criterion (FSC = 0.143)^44^ resulting in a final reconstruction of 2.9 Å (Supplementary Fig. 5b). Local-resolution estimation and local filtering of the final volumes were done using RELION-3.0^42^ (Supplementary Fig. 5c–e).

### Molecular modeling

The model of the *E. coli* 70S ribosome was derived from PDB ID 6TBV^45^. Domain-wise the proteins of the 30S and 50S and the rRNA were fitted separately into locally-filtered electron density maps using UCSF Chimera^46^ and Coot^47^. Afterwards manual adjustments were applied to all fitted molecular models using Coot. The ErmDL nascent chain was de novo modeled into the corresponding density. The final combined molecular models were then refined using the real space refinement procedure in Phenix version 1.14 together with restraints^48^. Statistics for the models were obtained using MolProbity^49^ and are represented in Supplementary Table 2.

### Figure preparation

Figures showing biochemical experiments are fitted and plotted with GraphPad Prism 8.0. Figures showing ribosome profiling data are created using R 3.3.1. Figures showing atomic models and electron densities were generated using either UCSF Chimera^46^ or Chimera X^50^ and assembled with Inkscape or Illustrator.

### Molecular dynamics (MD) simulations

To obtain the dynamics of ribosome-bound ErmDL in the presence and absence of the antibiotics ERY and TEL, we carried out all-atom explicit-solvent MD simulations of the region surrounding the peptide. For the simulation systems, all residues within 35 Å of the P-site tRNA CCA-end, the ErmDL peptide and the antibiotic were extracted. The residues of the system bound to residues not included were treated as described earlier^51^. The residues within a 25 Å radius were not restrained, while the outer-shell residues were subjected to position restraints with force constants obtained from their fluctuations in full-ribosome simulations as described earlier^51^. Three systems were simulated: one started from the cryo-EM structure with ERY (without A-site tRNA), one started from the cryo-EM structure with TEL (with A-site tRNA), and one from the ERY-structure after removal of ERY. For ion placement, Mg^2+^ ions with 5 Å of each simulation system were extracted from an aligned cryo-EM structure of the ribosome^52^. Histidine protonation states were determined using WHAT IF^53^. Next, each simulation system was first placed in a dodecahedron box with a minimum distance of 15 Å between the atoms and the box boundaries. Water molecules were added using the program solvate^54^.

The charge of the system was neutralized by iteratively replacing the water molecule with lowest Coulomb potential by a K^+^ ion. Subsequently, 7 mM MgCl_2_ and 150 mM KCl were added using the program GENION^54^. All simulations were carried out with GROMACS 2018^54^, the amber12 forcefield^55^, the SPC/E water model^56^, as well as K^+^ and Cl^−^ parameters from Joung and Cheatham^57^. Forcefield parameters for ERY were taken from Arenz et al.^28^ and parameters for TEL were obtained as described for ERY^28^. During the simulations, Lennard-Jones and short-range Coulomb interactions were calculated within a distance of 1 nm; long-range electrostatics were calculated using particle-mesh Ewald summation^58^. Bond lengths were constrained using the LINCS algorithm^59^ and virtual site constraints^60^ were used for hydrogen atoms, allowing an integration time step of 4 fs. Solute and solvent atoms were independently coupled to a heat bath at 300 K using velocity rescaling^61^ with a coupling constant of 0.1 ps. Initially, each system was energy-minimized (steepest decent) with harmonic position restraints applied to solute heavy atoms (*k* = 1000 kJ mol^−1^ nm^−1^). For each system, 20 sets of simulations were carried, each set consisting of three steps. First, the pressure was coupled to a Berendsen thermostat (coupling time 1 ps) and heavy-atom position restraints were applied for 50 ns. Second, during 20 ns, the position-restraints were linearly decreased to the force constant obtained from the full-ribosome simulations^51^ for the outer-shell and to zero for all other atoms. Finally, for 2-µs production simulations, the Parinello-Rahman barostat was used^62^. The 20 simulations for each of the three systems result in a total production simulation time of 120 µs. Coordinates were recorded every 5 ps.

### Assessing conformational changes, fluctuations and van-der-Waals clashes

At first, all trajectories were rigid-body fitted to the cryo-EM structure (+ERY) using the P atoms of 23S PTC nucleotides (746–748, 751–752, 789–790, 1614, 1781–1782, 2057–2059, 2061–2064, 2251–2253, 2439, 2450–2453, 2503–2508, 2553–2555, 2573, 2576, 2581, 2583–2586, 2601–2602, 2608–2612). Further, an X-ray structure of a *T. thermophilus* ribosome with a pre-attack A-site Phe-tRNA^Phe31^, a cryo-EM structure of an *E. coli* ribosome with an A-site Lys-tRNA^Lys28^, as well as two *H. marismortui* X-ray structures with the PTC in the uninduced and induced conformations^33,34^ were fitted to the cryo-EM structure in the same way. To compare the dynamics of Arg6 in the simulations with the two conformations resolved by cryo-EM in the presence of TEL, we calculated the probability density of the Arg6 Cζ atom using the GROmaps toolset with a *σ* of 0.5 Å and a spacing of 0.2 Å^63^. To investigate how the presence or absence of the antibiotics affects the accessible conformations of ErmDL, the extension of the peptide and its distance from the critical 23S rRNA nucleotide U2506 were monitored for all simulations. To monitor the peptide extension, the distance between the C3′ of the 3′ nucleotide of the P-site tRNA and the Cα atom of Thr2 were calculated. The distance from the A site was calculated between the Cα atom of Arg6 and the center of geometry of the centers of U2504 ribose atoms, U2506 phosphate atoms and the C2452 base atoms. For each set of simulations, the distances were sorted into 2-d bins and the logarithm of the probability $p={c}_{{{{\rm{i,j}}}}}/{c}_{{{{\rm{total}}}}}$ for each bin *i*, *j*, where ${c}_{{{{\rm{i,j}}}}}$ is the number of frames in the bin and ${c}_{{{{\rm{total}}}}}$ is the total number of frames was calculated.

To address the question to what extent the conformations of ErmDL interfere with the accommodation of an A-site tRNA, which entails nucleotides U2505 and U2585 adopting their induced conformations, we identified van-der-Waals clashes of the ErmDL peptide with residues from aligned structures. In particular, we identified clashes of Arg6 (ErmDL) with the Lys or Phe attached to the A-site tRNA^28,31^. Further, we identified clashes of Arg6 with an A-site Ala, obtained from the A-site Phe structure after removing side-chain atoms. Clashes with U2506 and U2585 were identified with ErmDL residues Arg6-His3 and His3-Thr2, respectively. To identify clashes, for each trajectory, we first extracted heavy-atom coordinates of the ErmDL residues at intervals of 50 ps. To find out if the ErmDL residues (set A with ${N}_{{{{\rm{A}}}}}$ atoms) clash with the aligned residues (set B with ${N}_{{{{\rm{B}}}}}$ atoms), for each of these snapshots, we constructed a rectangular box which contains the van-der-Waals spheres of all potentially overlapping atoms. To determine the box boundaries, we calculated the two minima, one, ${x}_{{\min },A}={{{\rm{min }}}}\left(\left\{{x}_{{{{\rm{i}}}}}-{r}_{{{{\rm{i}}}}},|,i\in \left\{1,\cdots ,{N}_{{{{\rm{A}}}}}\right\}\right\}\right)$, where ${x}_{{{{\rm{i}}}}}$ is the x-coordinate of atom $i$ from set A and ${r}_{{{{\rm{i}}}}}$ is its van-der-Waals radius, and, two, ${x}_{{\min },B}={{{\rm{min }}}}\left(\left\{{x}_{{{{\rm{j}}}}}-{r}_{{{{\rm{j}}}}},|,j\in \left\{1,\cdots ,{N}_{{{{\rm{B}}}}}\right\}\right\}\right)$ for atoms $j$ in set B. The maximum of the two minima is then used as the lower border of the box along the *x*-axis. The minimum of the two maxima along the x-axis determines the upper border of the box. For *y*-directions and *z*-directions, the borders were determined analogously. Next, a random coordinate within the box was drawn from a uniform distribution. If this coordinate was within the van-der-Waals radius of one atom in set A and of one atom in set B, the snapshot was considered clashing. Otherwise, iteratively, a new random coordinate was drawn until either a clash was found, or 50,000 coordinates were drawn. Only 250 ps intervals in which all five snapshots had an overlap of 0 were considered as non-overlapping conformations. To check which ErmDL conformations can resolve clashes, for each 2-d bin along the two distances (see above), we checked if there is a conformation that does not lead to a clash with A-site amino acids Lys, Phe, and Ala and the induced conformations of U2506 and U2585. To see for a lager set of A-site amino acids and amino acid conformations, in addition to the conformations obtained from structures (described above), if they clash with Arg6 conformations, we constructed all possible rotamer conformations of Val8, Ile8, Phe8, Met8, Lys8, Tyr8, Trp8, and Arg8. To that aim, we took all rotamer conformations from a rotamer library^64^, aligned them to the Lys or Phe attached to the A-site tRNA^28,31^ using the N-atoms, Cα-atoms, Cβ-atoms, and C-atoms. Next, we excluded the rotamer conformations which produced a van-der-Waals clash of heavy atoms with rRNA nucleotides. For the remaining conformations, we calculated van-der-Waals clashes with the Arg6 conformations observed in the simulations as described above.

In the simulations after removal of ERY, we observe conformations which do not overlap with the A-site Arg and the induced nucleotides. To check if resolving clashes is correlated with the ErmDL peptide moving into the space which would be occupied by ERY, for each frame of the −ERY simulations, two van-der-Waals overlap volumes were calculated. First, the overlap of the peptide with aligned ERY coordinates and, second, the overlap of the peptide with the A-site Lys and the induced nucleotides. Volumes were calculated as described for the clashes, except that all 50,000 coordinates were drawn. Volumes were estimated by multiplying the volume of the box with number of coordinates that are within van-der-Waals radii of one atom from set A and one atom from set B divided by 50,000.

### Reporting summary

Further information on research design is available in the Nature Research Reporting Summary linked to this article.

## Supplementary information

Supplementary Information

Reporting Summary

## Source data

Source Data

## Data Availability

The data that support this study are available from the corresponding authors upon reasonable request. The cryo-EM maps of the ErmDL-ERY-P-tRNA-SRC, ErmDL-ERY-A-P-tRNA-SRC and ErmDL-TEL-A-P-tRNA-SRC and the associated molecular models have been deposited in the Protein Data Bank and Electron Microscopy Data Bank with the accession codes EMD-12573, EMD-12574, EMD-12575, and PDB 7NSO, 7NSP, 7NSQ respectively. Sequencing data from the inverse toeprinting analysis have been deposited in the National Center for Biotechnology Information Short Read Archive with the accession code PRJNA623725. Source data are provided with this paper.
