# Supplementary material for: Structural and mechanistic basis for translation inhibition by macrolide and ketolide antibiotics
Source: Nat Commun. 2021 Jul 22;12:4466. doi: 10.1038/s41467-021-24674-9 (PMC8298421; doi:10.1038/s41467-021-24674-9)
Supplement: Supplementary file 1 — Supplementary Information [file 41467_2021_24674_MOESM1_ESM.pdf]

SUPPLEMENTARY DATA FOR

Structural and mechanistic basis for translation inhibition by macrolide and ketolide antibiotics

Bertrand Beckert[1,5], Elodie C. Leroy[2,5], Shanmugapriya Sotiselvam[3,5], Lars V. Bock[4,5,*], Maxim S. Svetlov[3], Michael Graf[1], Stefan Arenz[1], Maha Abdelshahid[1], Britta Seip[2], Helmut Grubmüller[4], Alexander S. Mankin[3], C. Axel Innis[2,*], Nora Vázquez-Laslop[3,*], and Daniel N. Wilson[1,*]

[1] Institute for Biochemistry and Molecular Biology, University of Hamburg, 20146 Hamburg, Germany.
[2] Univ. Bordeaux, Centre National de la Recherche Scientifique, Institut National de la Santé et de la Recherche Médicale, ARNA, UMR 5320, U1212, Institut Européen de Chimie et Biologie, F-33600 Pessac, France.
[3] Center for Biomolecular Sciences, University of Illinois at Chicago, Chicago, IL 60607
[4] Theoretical and Computational Biophysics Department, Max Planck Institute for Biophysical Chemistry, Am Fassberg 11, 37077, Göttingen, Germany.

[5] These authors contributed equally

*Correspondence: daniel.wilson@chemie.uni-hamburg.de, axel.innis@inserm.fr, nvazquez@uic.edu and lbock@mpibpc.mpg.de

Supplementary Notes

A previous study examining the macrolide-dependent stalling mechanism of a short MRLR sequence also concluded that in addition to the length, charge also plays an important role[1]. A comparison of their results in light of our structural data illustrates an excellent correspondence. On the one hand, the length of the side chain of the aminoacyl-moiety of the CCA-end mimic played an important role since L-norleucine, which has a 4-carbon side chain (i.e. shorter than Lys or Arg), reacted rapidly with the MRL-tRNA independent of the presence of ERY, analogously to L-Ala[1]. By contrast, extending the side chain by an additional one or two atoms as in 6-hydroxy-L-norleucine or 5-ethyl-L-norleucine, respectively, led to a >6-fold reduction in the rate of peptide bond formation[1]. Consistently, modeling L-norleucine into the A-site of the ErmDL-TEL-SRC indicates that the clash of L-norleucine with Arg6 of ErmDL is less than for 6-hydroxy-L-norleucine or 5-ethyl-L-norleucine (**Supplementary Fig. 4e-g**). However, one the other hand, the rates of 6-hydroxy-L-norleucine and 5-ethyl-L-norleucine were still 30-fold slower than Lys or Arg[1], which have the same side chain lengths, respectively, suggesting that charge makes an important contribution. Indeed, L-ornithine, which is the same length as L-norleucine (and therefore displays only modest overlap with Arg6 of ErmDL, **Supplementary Fig. 4e**), but carries a positive charge, reacts with similar rates as Arg and Lys[1]. Finally, the positively charged L-aminoalanine, which barely overlaps with Arg6 of ErmDL (**Supplementary Fig. 4h**), also displayed a 60-fold reduction in rate compared to Arg and Lys[1].

Supplementary Figures

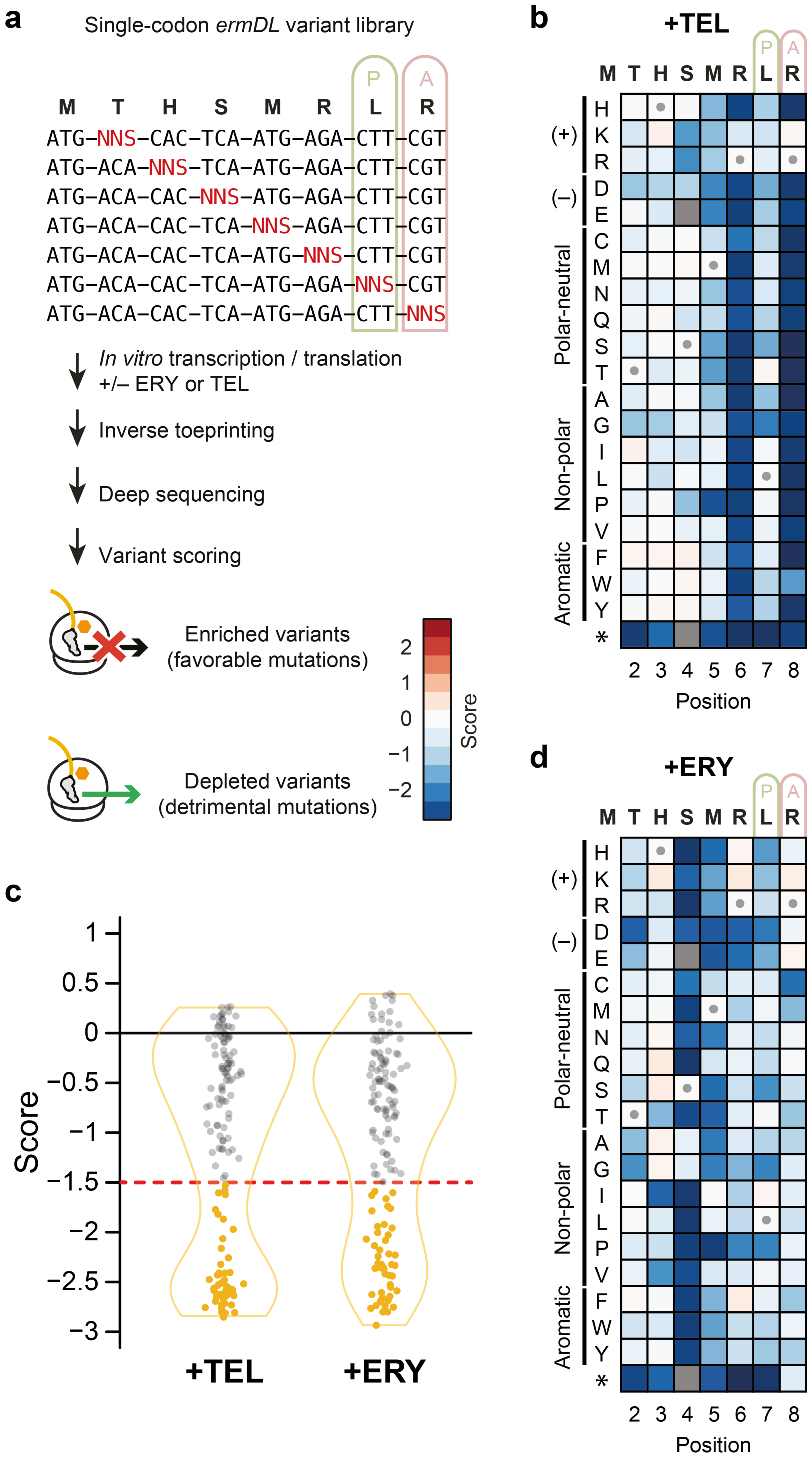

Supplementary Figure 1: Deep mutational scans of ErmDL in the presence of TEL or ERY. a A library of single-codon *ermDL* variants was translated *in vitro* in the absence or presence of 50 µM telithromycin (TEL) or 50 µM erythromycin (ERY), and subjected to inverse toeprinting[2] followed by deep sequencing. Sequencing reads were quality filtered and variants were counted with Enrich2[3]. **b** Sequence-function map for 7 positions of the ErmDL peptide translated in the presence of TEL. Cell color indicates the score for a single amino acid change at a given position. Positive scores (red) indicate better than wild-type translational

arrest and negative scores (blue) indicate worse than wild-type performance. The ribosomal P (green) and A (pink) sites are indicated above the wild-type ErmDL sequence. Variants in grey were not measured and cells marked with a circle correspond to the wild-type amino acid. **c** RDI (raw data, description and inference) plot showing Enrich2[3] score distributions for single-codon *ermDL* variants translated in the absence or presence of 50 μM TEL or 50 μM ERY. The red dashed line corresponds to the -1.5 score threshold used to identify deleterious variants (yellow). Note that all mutations have worse than wild-type performance and that variant scores follow a bimodal distribution in each case. **d** Sequence-function map for 7 positions of the ErmDL peptide translated in the presence of ERY. Colors are the same as in **b**.

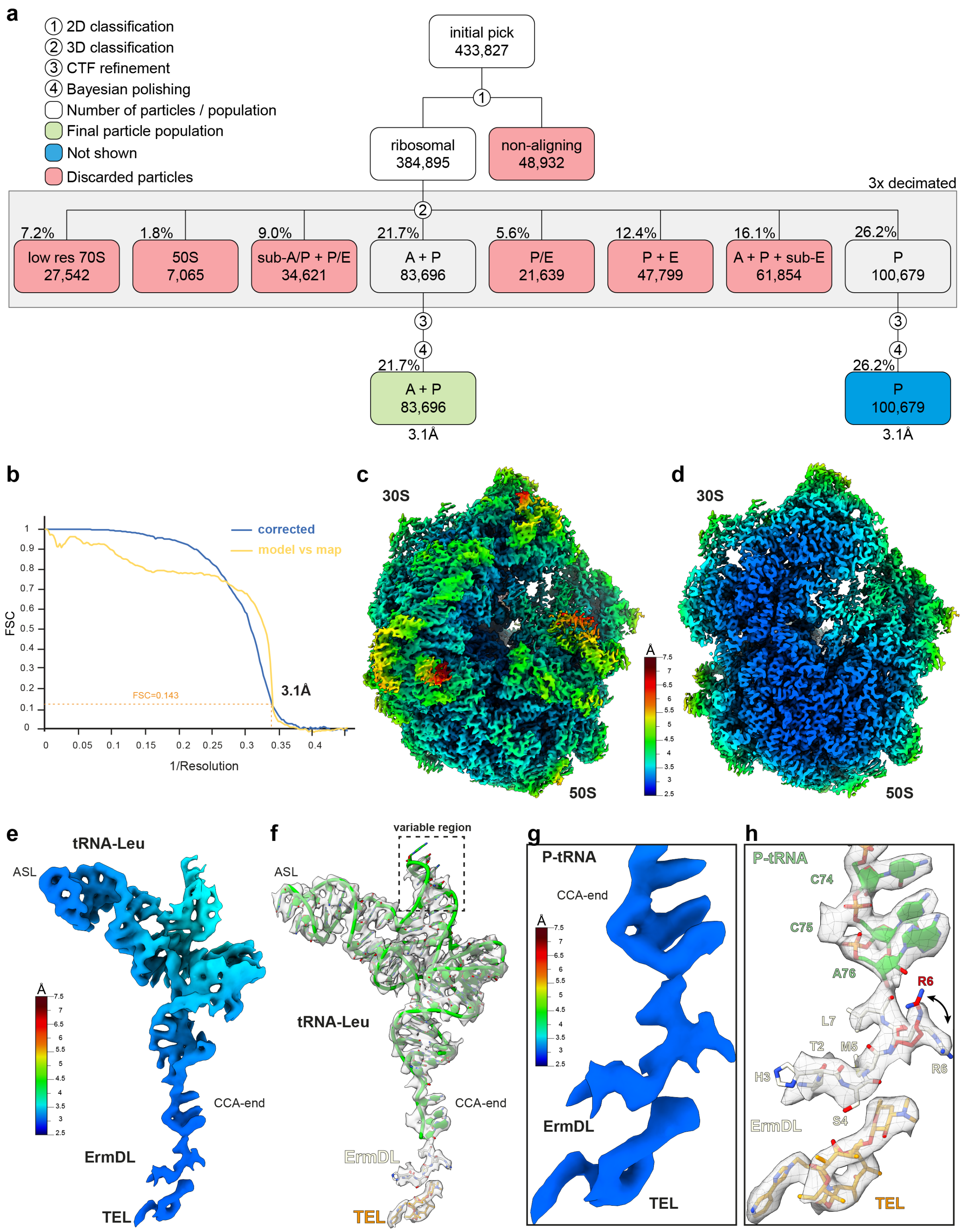

Supplementary Figure 2 Processing of the cryo-EM data of ErmDL-TEL-SRC. a Sorting scheme for processing of the ErmDL-TEL-SRC. **b** Fourier Shell Correlation (FSC) corrected (blue) and map vs model (yellow) curves with the resolution at FSC=0.143 indicated by a dashed line. **c** Overview and **d** transverse section of the ErmDL-TEL-SRC coloured according to local resolution. **e-h** Isolated densities for the ErmDL-tRNA and TEL **e,g** colored according to local resolution and **f, h** shown as mesh with fitted model.

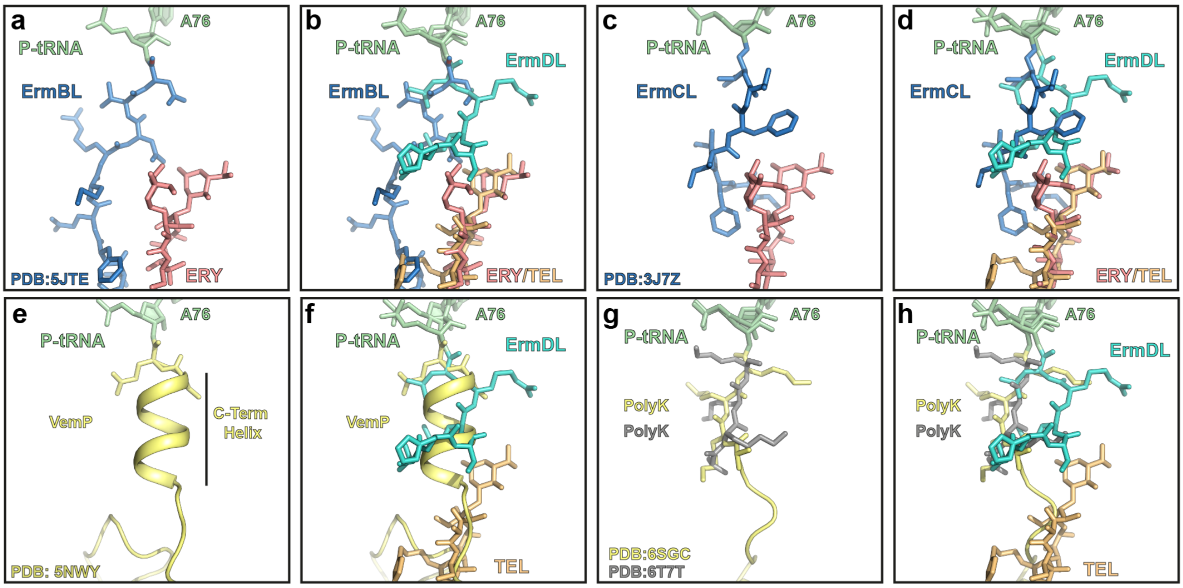

Supplementary Figure 3: Comparison of ErmDL with other nascent polypeptide chains. **a-f** The path and conformation of **a** ErmBL (blue, PDB ID 5JTE)[4] superimposed with **b** ErmDL (cyan), **c** ErmCL (blue, PDB ID 3J7Z)[5] superimposed with **d** ErmDL (cyan), **e** VemP (yellow, PDB ID 5NWY)[6] superimposed with **f** ErmDL (cyan), and **g** poly(Lys) nascent chains (yellow, PDB ID 6SGC; gray, PDB ID 6T7T)[7,8] superimposed with **h** ErmDL (cyan).

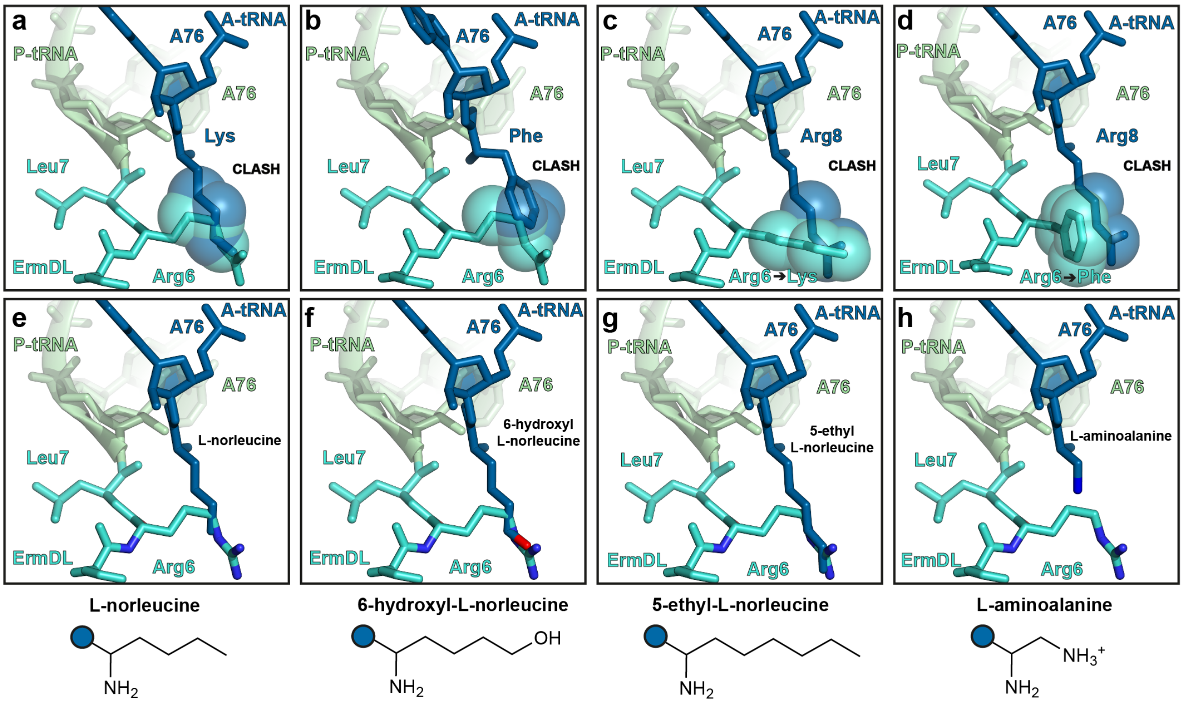

Supplementary Figure 4: Superimposition of Arg6 of ErmDL with different A-site tRNAs. a-b Superimposition of the ErmDL-tRNA in the P-site of the ErmDL-TEL-SRC with an A-site **a** Lys-tRNA from ErmBL (blue, PDB ID 5JTE)[4] or **b** Phe-tRNA from the pre-attack state (blue, PDB ID 1VY7)[9], illustrating that Arg6 of ErmDL nascent chain clashes (indicated by spheres) with the Lys and Phe side chains of the accommodated A-site tRNA, respectively. **c-d** *In silico* mutations of Arg6 of ErmDL to **c** Lys or **d** Phe, illustrating clashes with the arginyl moiety of an Arg8-tRNA accommodated at the A-site. **e-h** Superimposition of the ErmDL-tRNA in the P-site of the ErmDL-TEL-SRC with an A-site CCA-end mimic bearing **e** L-norleucine, **f** 6-hydroxy-L-norleucine, **g** 5-ethyl-L-norleucine, or **h** L-aminoalanine, with respective chemical structures of the non-canonical amino acids shown below each respective panel. Models for the non-canonical amino acids were generated by mutation of the Phe-tRNA from the pre-attack state (blue, PDB ID 1VY7)[9].

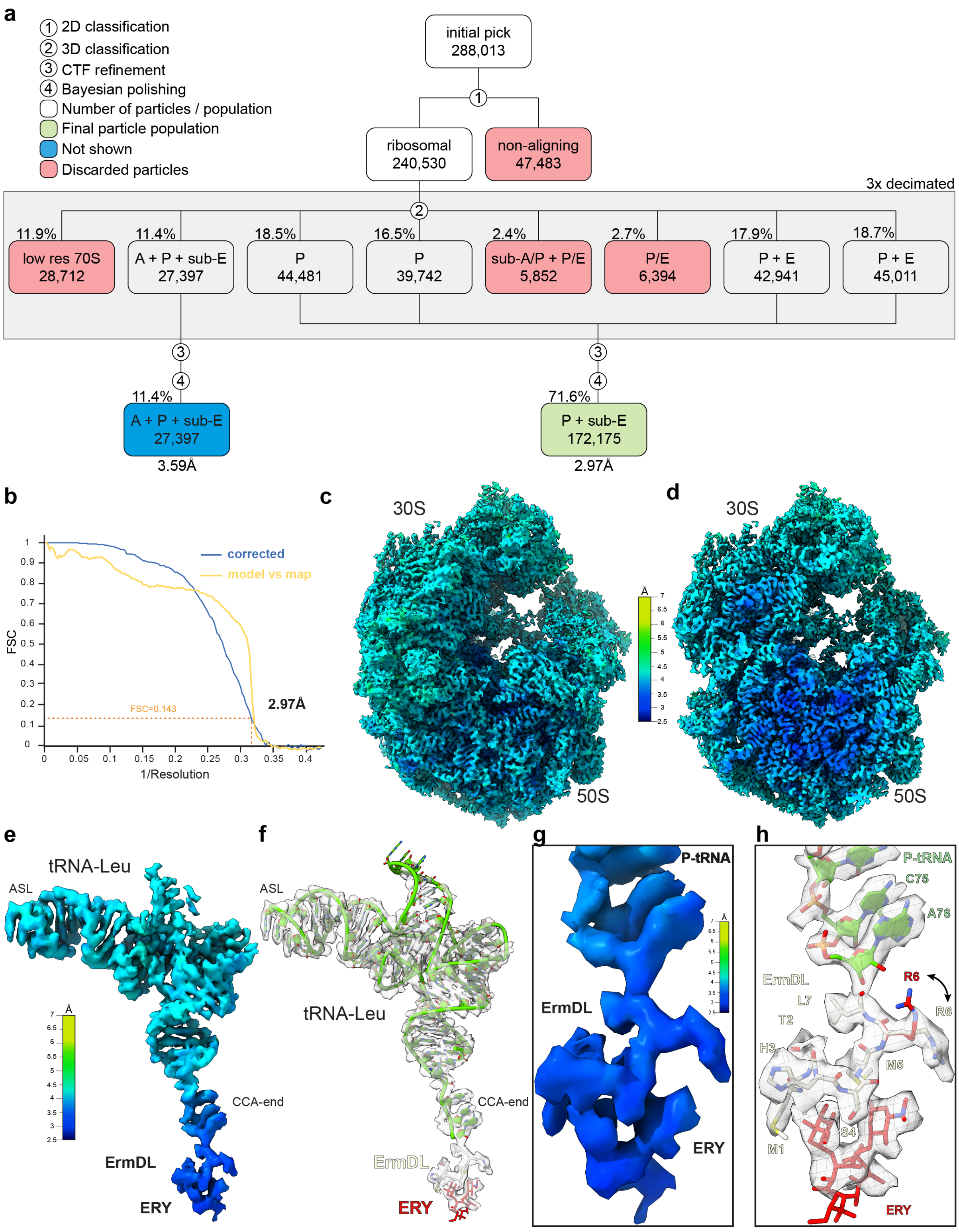

Supplementary Figure 5: Processing of the cryo-EM data of ErmDL-ERY-SRC. a Sorting scheme for processing of the ErmDL-ERY-SRC. **b** Fourier Shell Correlation (FSC) corrected (blue) and map vs model (yellow) curves with the resolution at FSC=0.143 indicated by a dashed line. **c** Overview and **d** transverse section of the ErmDL-ERY-SRC coloured according to local resolution. **e-h** Isolated densities for the ErmDL-tRNA and ERY **e,g** colored according to local resolution and **f, h** shown as mesh with fitted model.

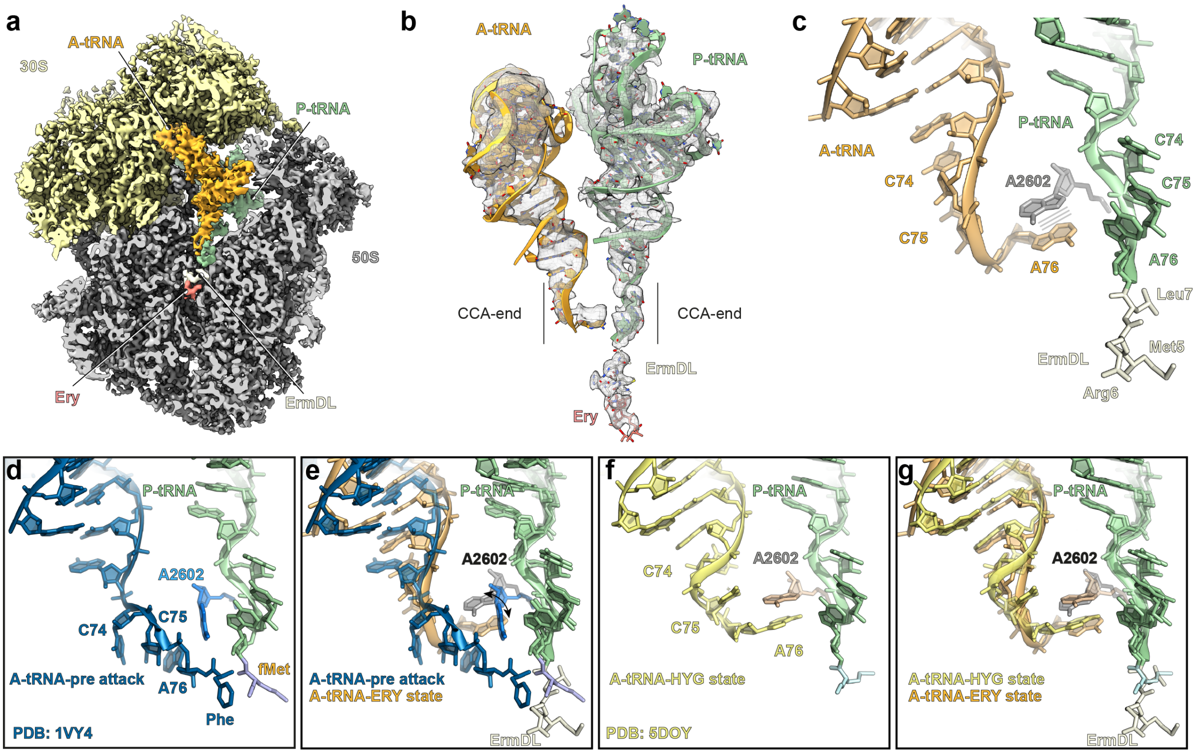

Supplementary Figure 6: Conformation of A-site tRNA in the ErmDL-ERY-SRC. a Transverse section of the cryo-EM map of the ErmDL-ERY-SRC with isolated densities highlighting the 30S (yellow) and 50S (grey) subunits, P-site tRNA (green), A-site tRNA (orange), ErmDL nascent chain (beige) and ERY (salmon). **b** Isolated densities (grey mesh) for the A-site tRNA (orange), ErmDL-tRNA (green) and ERY (red). **c** Molecular model of the A-site tRNA (orange), ErmDL nascent chain (beige), P-site tRNA (green) and erythromycin (ERY, red). **d-g** Conformation of the **d** A-site tRNA from the ErmDL-ERY-SRC compared to (**e**) pre-attack A-site tRNA (PDB 1VY4) and **f, g** A-site tRNA (yellow) in the presence of hygromycin A (PDB 5DOY).

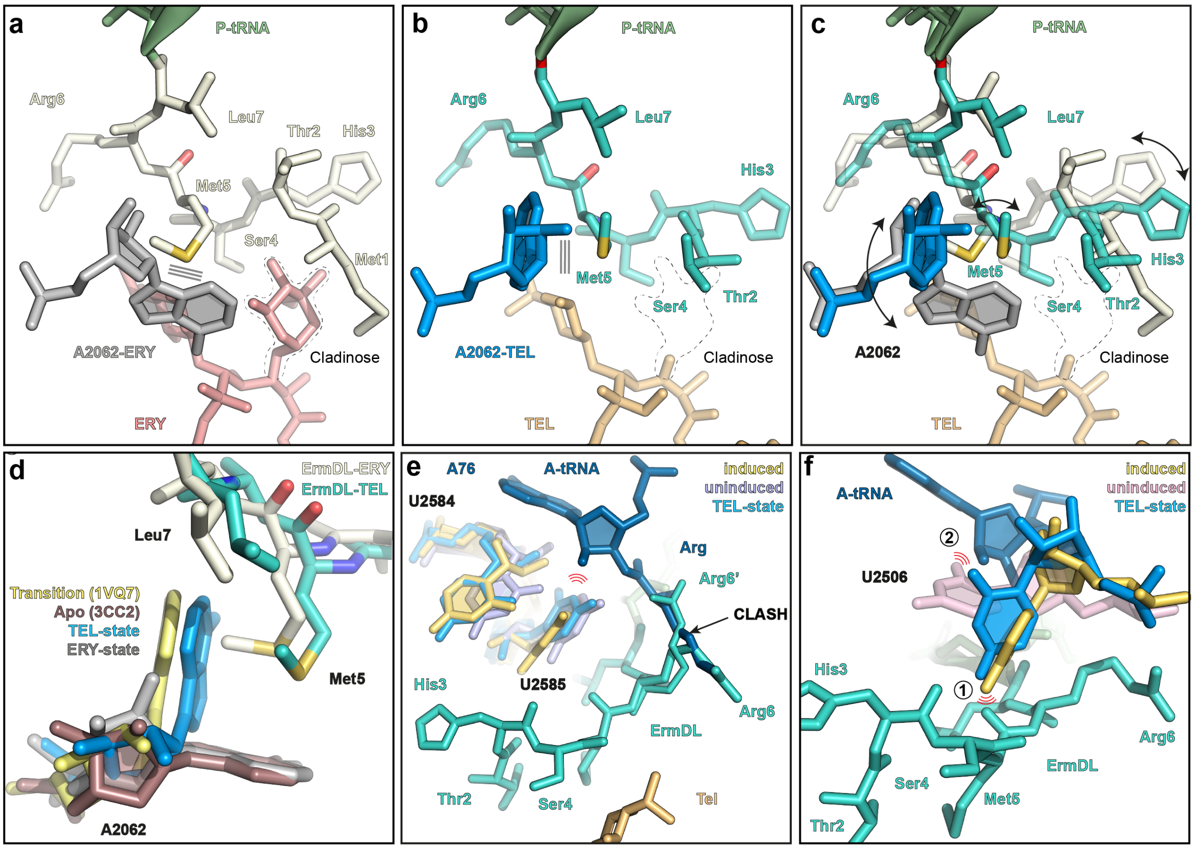

Supplementary Figure 7: Interactions and conformational changes within the PTC of the ErmDL-ERY-SRC. a-c Comparison of the conformation of A2062 in **a** ErmDL-ERY-SRC and **b** ErmDL-TEL-SRC, with **c** superimposition of **a** and **b** showing the rotation of A2062 (arrowed). **d** Comparison of the conformation of A2062 in ErmDL-ERY-SRC (gray) with ErmDL-ERY-SRC (blue), transition state (yellow, PDB 1VQ7)[10] and Apo state (violet, PDB 3CC2)[11]. **e-f** Comparison of the conformation of **e** U2584 and U2585 and **f** U2506 in ErmDL-TEL-SRC (blue) with the uninduced (pink, PDB 1VQN) and induced states (yellow, PDB 1VQ6)[10].

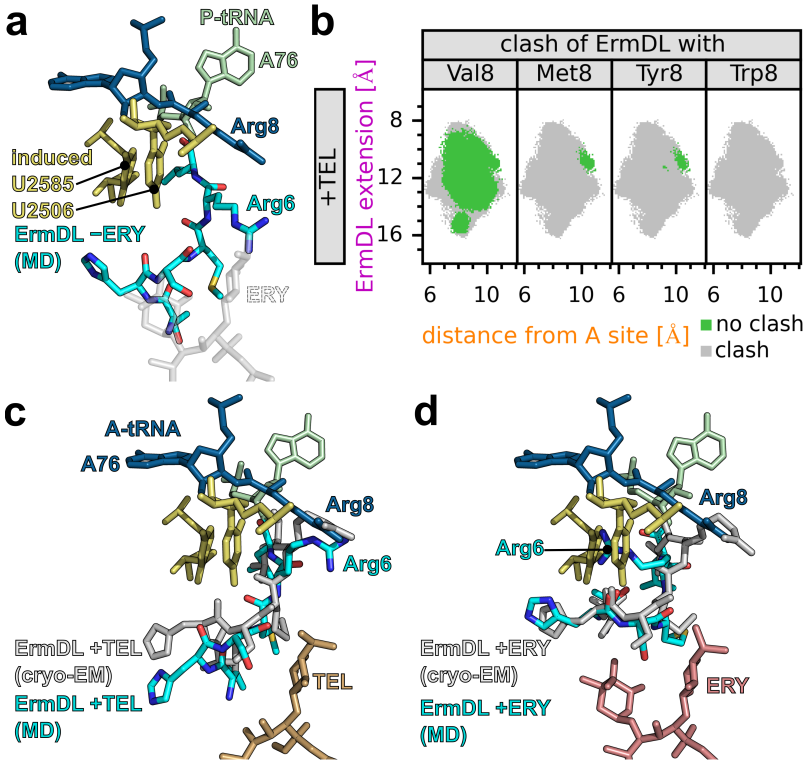

Supplementary Figure 8: Dynamics of ErmDL obtained from MD simulations. a, c, d Conformations ErmDL and antibiotics obtained from MD simulations (cyan) and cryo-EM (grey) along with A76 and Arg8 of the aligned A-tRNA (blue) and with the induced conformations of U2506 and U2585[10,12] (yellow) are shown. Conformations selected from the MD simulations: after removal of ERY, a conformation not clashing with Arg8 and the induced nucleotides **a**, in the presence of TEL, an ErmDL conformation that does not clash with Ala8 and with induced conformations of U2506 and U2585 **c**, and in the presence of ERY, a conformation not clashing with Arg8 **d**. **b** The ErmDL conformations observed in the MD simulations (+TEL) are shown (compare Fig. 6B). For all rotamer conformations of A-site amino acids (Val8, Met8, Tyr8, Trp8), van der Waals clashes with ErmDL were calculated. Green areas indicate conformations which do not clash with at least one rotamer conformation of the A-site amino acid.

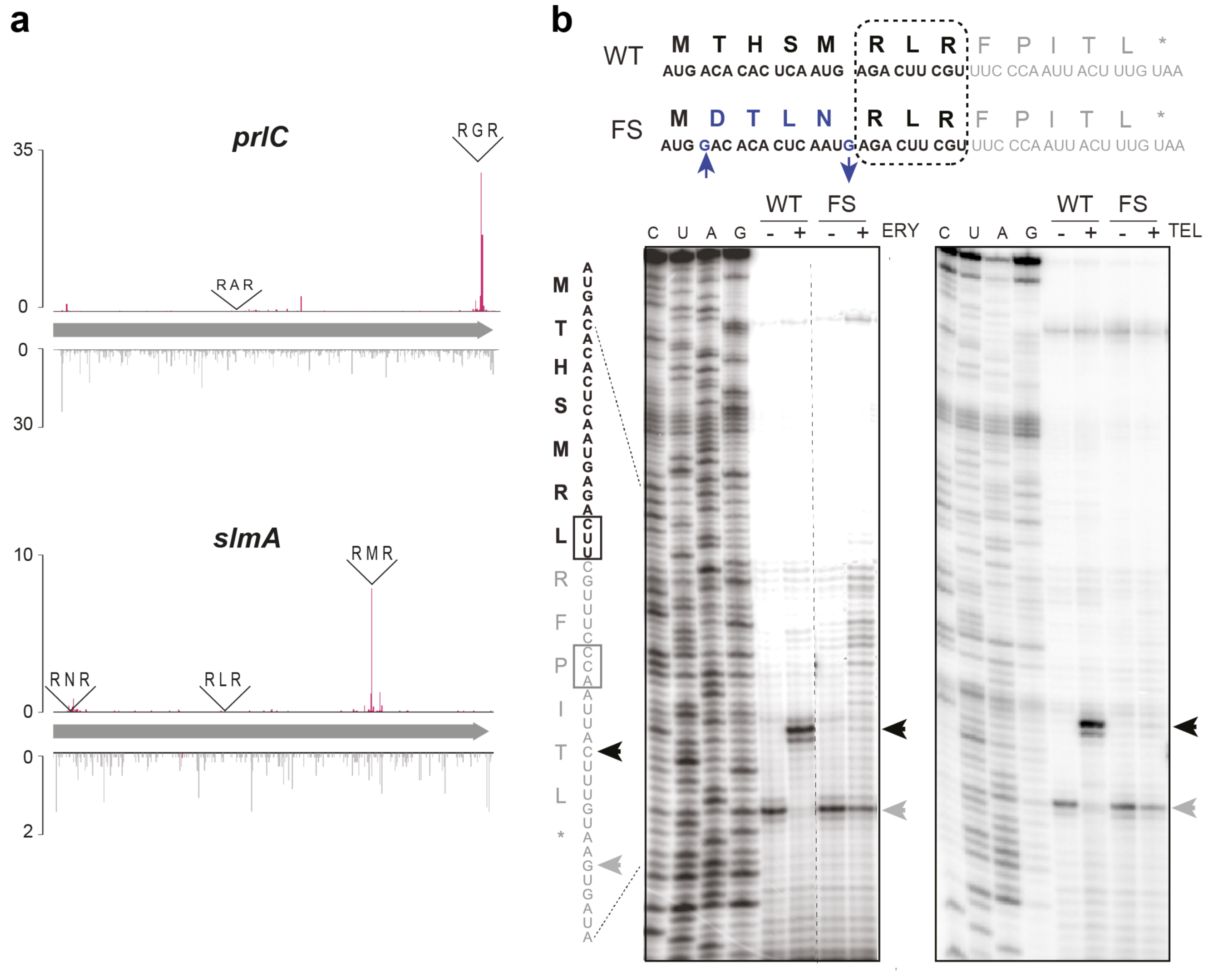

Supplementary Figure 9: Context affects stalling at +X+ motifs. a Ribo-seq profiles of the open reading frames of the *prlC* and *slmA* genes of *E. coli* cells untreated (ribosomal footprints density peaks in gray) or treated with TEL (density peaks in red). The Ribo-seq profiles were taken from Kannan et al., 2014. The profile of *prlC* shows that TEL-bound ribosomes translated through the early Arg-Ala-Arg (+X+) sequence but then became arrested at the Arg-Gly-Arg (+X+) motif towards the end of the ORF. Similarly, TEL failed to induce stalling at the +X+ sequences Arg-Asn-Arg and Arg-Leu-Arg of *slmA* but successfully prompted arrest at the downstream Arg-Met-Arg sequence. **b** *Top*: The sequence of the *ermDL* ORF (WT) or its mutant variant (FS) where the identities of the N-terminal amino acid residues preceding the arrest motif were modified by single-nucleotide indel compensatory mutations (indicated by blue arrows). *In vitro* toeprinting analysis testing the ERY- and TEL- mediated translation arrest at the Arg-Leu-Arg (+X+) motif of the *ermDL* ORF (WT) or its mutant variant (FS). The toeprint bands of ERY or TEL stalled ribosomes with the Leu7 codon of the WT ORF at the P site are indicated by black arrows. The toeprint bands of ribosomes stalled at codon 10 due to the lack of charged Ile-tRNA (because of the presence of mupirocin in the reactions) are shown with gray arrows. Sequencing reactions are labeled as C, U, A, G. Toeprinting was performed in duplicate and representative gel is shown.

Supplementary Tables

Supplementary Table 1: Primers used in this work

Primer	Sequence
ermDL-NdeI-fwd	5'-GATAACATATGACACACTCAATGAGAC-3'
ermD-AflII-rev	5'-TTATCCTTAAGATGATTTTTTTCTTCATGTTTCC-3'
T7-MTHSM-fwd	5'-TAATACGACTCACTATAGGGCTTAAGTATAAGGAGGAAAAAATATGACACACTCAATG-3'
NV1-IFPTL-rev	5'-GGTTATAATGAATTTTGCTTATTAACGATAGAATTCTATCACTTACAAAGTTGGGAAAAT-3'
RLR-IFPTL-fwd	5'-GGAGGAAAAAATATGACACACTCAATGAGACTTCGTATTTTCCC-3'
ALR-IFPTL-fwd	5'-GGAGGAAAAAATATGACACACTCAATGGCACTTCGTATTTTCCC-3'
RLA-IFPTL-fwd	5'-GGAGGAAAAAATATGACACACTCAATGAGACTTGCAATTTTCCC-3'
ALA-IFPTL-fwd	5'-GGAGGAAAAAATATGACACACTCAATGGCACTTGCAATTTTCCC-3'
AAA-IFPTL-fwd	5'-GGAGGAAAAAATATGACACACTCAATGGCAGCAGCAATTTTCCC-3'
RLR-IFPTL-rev	5'-CTATCACTTACAAAGTTGGGAAAATACGAAGTCTCATTGAG-3'
ALR-IFPTL-rev	5'-CTATCACTTACAAAGTTGGGAAAATACGAAGTGCCATTGAG-3'
RLA-IFPTL-rev	5'-CTATCACTTACAAAGTTGGGAAAATTGCAAGTCTCATTGAG-3'
ALA-IFPTL-rev	5'-CTATCACTTACAAAGTTGGGAAAATTGCAAGTGCCATTGAG-3'
AAA-IFPTL-rev	5'-CTATCACTTACAAAGTTGGGAAAATTGCTGCTGCCATTGAG-3'
T7	5'-TAATACGACTCACTATAGGG -3'
NV1	5'-GGTTATAATGAATTTTGCTTATTAAC -3'
M3-MDTLN-fwd	5'-TAATACGACTCACTATAGGGCTTAAGTATAAGGAGGAAAAAATATGGACACACTCAATAGACTTCGTTTCCCAATTACTTTGTAAGTG -3'
WT-MTHSM-fwd	5'-TAATACGACTCACTATAGGGCTTAAGTATAAGGAGGAAAAAATATGACACACTCAATGAGACTTCGTTTCCCAATTACTTTGTAAGTG-3'
ermD-58 nts-rev	5'-GGTTATAATGAATTTTGCTTATTAACGATAGAATTCTATCACTTACAAAGTAATTGGG -3'
T7_RBS_ATG_f	5'-CGATCGAATTCTAATACGACTCACTATAGGGCTTAAGTATAAGGAGGAAAAAATATG-3'
Stop_EcoRV_r	5'-TATATGGATCCTTTTTGATATTGATATCTCATCACACCGAGATCG-3'
T7_f	5'-CGATCGAATTCTAATACGACTCACTATAG-3'
EcoRV_r	5'-TATATGGATCCTTTTTGATATTGATA-3'
ermDL-NNS2	5'-GGAGGAAAAAATATGNNSCACTCAATGAGACTTCGTGCGATCTCGGTGTGAT-3'
ermDL-NNS3	5'-GGAGGAAAAAATATGACANNSTCAATGAGACTTCGTGCGATCTCGGTGTGAT-3'
ermDL-NNS4	5'-GGAGGAAAAAATATGACACACNNSATGAGACTTCGTGCGATCTCGGTGTGAT-3'
ermDL-NNS5	5'-GGAGGAAAAAATATGACACACTCANNSAGACTTCGTGCGATCTCGGTGTGAT-3'
ermDL-NNS6	5'-GGAGGAAAAAATATGACACACTCAATGNNSCTTCGTGCGATCTCGGTGTGAT-3'
ermDL-NNS7	5'-GGAGGAAAAAATATGACACACTCAATGAGANNSCGTGCGATCTCGGTGTGAT-3'
ermDL-NNS8	5'-GGAGGAAAAAATATGACACACTCAATGAGACTTNNSGCGATCTCGGTGTGAT-3'
3'_linker_ApoI	5'-/5rAPP/GGTATCTCGGTGTGACTGACTGAAAATTTCTGTAGGCACCATCAAT/ddC-3'
Linker_r	5'-ATTGATGGTGCCTACAG-3'
cDNA_f	5'-GTATAAGGAGGAAAAAATATG-3'
Biotin_standard	5'-/5Biosg/AAAAAAAAAAAAAATTAACTCCATCTAA-3'
NGS_f	5'-AATGATACGGCGACCACCG-3'

NGS_r	5'-CAAGCAGAAGACGGCATACGAG-3'
NGS_adapter_f	5'-AATGATACGGCGACCACCGAGATCTACACTCTTTCCCTACACGACGCTCTTCCGATCTG TATAAGGAGGAAAAAATATG-3'
NGS_adapter_ind ex23	5'-CAAGCAGAAGACGGCATACGAGATATCCACTCCTGTGACTGGAGTTCAGACGTGTGCT CTTCCGATCGATTGATGGTGCCTACAG-3'
NGS_adapter_ind ex25	5'-CAAGCAGAAGACGGCATACGAGATATATCAGTCTGTGACTGGAGTTCAGACGTGTGCT CTTCCGATCGATTGATGGTGCCTACAG-3'
NGS_adapter_ind ex27	5'-CAAGCAGAAGACGGCATACGAGATAAAGGAATCTGTGACTGGAGTTCAGACGTGTGCT CTTCCGATCGATTGATGGTGCCTACAG-3'
NGS_adapter_ind ex28	5'-CAAGCAGAAGACGGCATACGAGATCTTTTGGTGACTGGAGTTCAGACGTGTGCTCTTC CGATCGATTGATGGTGCCTACAG-3'
NGS_adapter_ind ex29	5'-CAAGCAGAAGACGGCATACGAGATTAGTTGGTGACTGGAGTTCAGACGTGTGCTCTTC CGATCGATTGATGGTGCCTACAG-3'
NGS_adapter_ind ex30	5'-CAAGCAGAAGACGGCATACGAGATCCGGTGGTGACTGGAGTTCAGACGTGTGCTCTTC CGATCGATTGATGGTGCCTACAG-3'
NGS_adapter_ind ex31	5'-CAAGCAGAAGACGGCATACGAGATATCGTGGTGACTGGAGTTCAGACGTGTGCTCTTC CGATCGATTGATGGTGCCTACAG-3'
NGS_adapter_ind ex32	5'-CAAGCAGAAGACGGCATACGAGATTGAGTGGTGACTGGAGTTCAGACGTGTGCTCTTC CGATCGATTGATGGTGCCTACAG-3'
NGS_adapter_ind ex36	5'-CAAGCAGAAGACGGCATACGAGATTGTTGGGTGACTGGAGTTCAGACGTGTGCTCTTC CGATCGATTGATGGTGCCTACAG-3'
NGS_adapter_ind ex39	5'-CAAGCAGAAGACGGCATACGAGATGTATAGGTGACTGGAGTTCAGACGTGTGCTCTTC CGATCGATTGATGGTGCCTACAG-3'
NGS_adapter_ind ex40	5'-CAAGCAGAAGACGGCATACGAGATTCTGAGGTGACTGGAGTTCAGACGTGTGCTCTTC CGATCGATTGATGGTGCCTACAG-3'
NGS_adapter_ind ex41	5'-CAAGCAGAAGACGGCATACGAGATGTCGTCGTGACTGGAGTTCAGACGTGTGCTCTTC CGATCGATTGATGGTGCCTACAG-3'
NGS_adapter_ind ex42	5'-CAAGCAGAAGACGGCATACGAGATCGATTAGTGACTGGAGTTCAGACGTGTGCTCTTC CGATCGATTGATGGTGCCTACAG-3'
NGS_adapter_ind ex43	5'-CAAGCAGAAGACGGCATACGAGATGCTGTAGTGACTGGAGTTCAGACGTGTGCTCTTC CGATCGATTGATGGTGCCTACAG-3'
NGS_adapter_ind ex44	5'-CAAGCAGAAGACGGCATACGAGATATTATAGTGACTGGAGTTCAGACGTGTGCTCTTC CGATCGATTGATGGTGCCTACAG-3'
NGS_adapter_ind ex19+3	5'-CAAGCAGAAGACGGCATACGAGATTTTCACGTGACTGGAGTTCAGACGTGTGCTCTTC CGATCCTCGATTGATGGTGCCTACAG-3'
NGS_adapter_ind ex21+1	5'-CAAGCAGAAGACGGCATACGAGATCGAAACGTGACTGGAGTTCAGACGTGTGCTCTTC CGATCAGATTGATGGTGCCTACAG-3'
NGS_adapter_ind ex23+3	5'-CAAGCAGAAGACGGCATACGAGATCCACTCGTGACTGGAGTTCAGACGTGTGCTCTTC CGATCCTCGATTGATGGTGCCTACAG-3'

Supplementary Table 2: Cryo-EM data collection and model refinement statistics:

**Data Collection and Refinement**	**ErmDL-ERY P-tRNA-SRC** PDB ID: 7NSO EMDB: 12573	**ErmDL-ERY A-P-tRNA-SRC** PDB ID: 7NSP EMDB: 12574	**ErmDL-TEL A-P-tRNA-SRC** PDB ID: 7NSQ EMDB: 12575
Particles	172,175	27,397	83,696
Pixel size (Å)	1.108	1.108	1.084
Defocus range (µm)	-0.7 to -3	-0.7 to -3	-0.7 to -3
Voltage (kV)	300	300	300
Magnification	129,151	129,151	129,151
Electron dose ($e^-/Å^2$)	2.8	2.8	2.5
Camera	Falcon II	Falcon II	Falcon II
**Model Composition**			
Protein residues	5,684	5,684	5,684
RNA bases	4,714	4,733	4,733
**Refinement**			
Initial model used (PDB code)	6TBV	6TBV	6TBV
Resolution (Å) $FSC_{0.143}$	2.97	3.5	3.12
Map Resolution Range	2.3-7	2.5-7	2.5-7
Map sharpening B factor ($Å^2$)	-61.80	-53.68	-62.05
Box size	360^3	360^3	360^3
**Validation Proteins**			
Poor rotamers (%)	0.69	0.95	0.54
Ramachandran outliers (%)	0.29	0.30	0.3
Ramachandran favored (%)	92.61	91.58	92.93
Bad backbone angles (%)	0.07	0.07	0.06
**Validation RNA**			
Correct sugar puckers (%)	98.71	98.75	98.82
Good backbone conformations (%)	78.4	76.76	78.83
Bad angles (%)	0.04	0.07	0.03
**Scores**			
MolProbity score	1.68	1.80	1.66
Clash score, all atoms	4.38	5.5	4.3

Supplementary References

1 Sothiselvam, S. *et al.* Binding of Macrolide Antibiotics Leads to Ribosomal Selection against Specific Substrates Based on Their Charge and Size. *Cell reports* **16**, 1789-1799, doi:10.1016/j.celrep.2016.07.018 (2016).

2 Seip, B., Sacheau, G., Dupuy, D. & Innis, C. A. Ribosomal stalling landscapes revealed by high-throughput inverse toeprinting of mRNA libraries. *Life Sci Alliance* **1**, e201800148, doi:10.26508/lsa.201800148 (2018).

3 Rubin, A. F. *et al.* A statistical framework for analyzing deep mutational scanning data. *Genome biology* **18**, 150, doi:10.1186/s13059-017-1272-5 (2017).

4 Arenz, S. *et al.* A combined cryo-EM and molecular dynamics approach reveals the mechanism of ErmBL-mediated translation arrest. *Nature communications* **7**, 12026 (2016).

5 Arenz, S. *et al.* Molecular basis for erythromycin-dependent ribosome stalling during translation of the ErmBL leader peptide. *Nat Commun* **5**, 3501, doi:10.1038/ncomms4501 (2014).

6 Su, T. *et al.* The force-sensing peptide VemP employs extreme compaction and secondary structure formation to induce ribosomal stalling. *eLife* **6**, doi:10.7554/eLife.25642 (2017).

7 Chandrasekaran, V. *et al.* Mechanism of ribosome stalling during translation of a poly(A) tail. *Nat Struct Mol Biol* **26**, 1132-1140, doi:10.1038/s41594-019-0331-x (2019).

8 Tesina, P. *et al.* Molecular mechanism of translational stalling by inhibitory codon combinations and poly(A) tracts. *EMBO J* **39**, e103365, doi:10.15252/embj.2019103365 (2020).

9 Polikanov, Y. S., Steitz, T. A. & Innis, C. A. A proton wire to couple aminoacyl-tRNA accommodation and peptide-bond formation on the ribosome. *Nat Struct Mol Biol* **21**, 787-793, doi:10.1038/nsmb.2871 (2014).

10 Schmeing, T. M., Huang, K. S., Strobel, S. A. & Steitz, T. A. An induced-fit mechanism to promote peptide bond formation and exclude hydrolysis of peptidyl-tRNA. *Nature* **438**, 520-524 (2005).

11 Blaha, G., Gurel, G., Schroeder, S. J., Moore, P. B. & Steitz, T. A. Mutations outside the anisomycin-binding site can make ribosomes drug-resistant. *J. Mol. Biol.* **379**, 505-519 (2008).

12 Schmeing, T. M., Huang, K. S., Kitchen, D. E., Strobel, S. A. & Steitz, T. A. Structural insights into the roles of water and the 2' hydroxyl of the P site tRNA in the peptidyl transferase reaction. *Mol. Cell* **20**, 437-448 (2005).
